# The Structural Basis for Optimal Performance of Oligothiophene-Based Fluorescent Amyloid Ligands: Conformational Flexibility is Essential for Spectral Assignment of a Diversity of Protein Aggregates

**DOI:** 10.1002/chem.201301463

**Published:** 2013-06-18

**Authors:** Therése Klingstedt, Hamid Shirani, K O Andreas Åslund, Nigel J Cairns, Christina J Sigurdson, Michel Goedert, K Peter R Nilsson*

**Affiliations:** [a] Department of Chemistry, Linköping University581 83 Linköping (Sweden) E-mail: petni@ifm.liu.se; [b] Department of Neurology, Alzheimer–s Disease Research Center, Washington UniversitySt. Louis, Missouri 63110 (USA); [c] Department of Pathology, University of CaliforniaSan Diego, La Jolla, California 92093-0612 (USA); [d] MRC Laboratory of Molecular BiologyHills Road, Cambridge CB2 0QH (UK)

**Keywords:** Alzheimer’s disease, fluorescent probes, luminescent conjugated oligothiophenes, microscopy, protein folding

## Abstract

Protein misfolding diseases are characterized by deposition of protein aggregates, and optical ligands for molecular characterization of these disease-associated structures are important for understanding their potential role in the pathogenesis of the disease. Luminescent conjugated oligothiophenes (LCOs) have proven useful for optical identification of a broader subset of disease-associated protein aggregates than conventional ligands, such as thioflavin T and Congo red. Herein, the molecular requirements for achieving LCOs able to detect nonthioflavinophilic Aβ aggregates or non-congophilic prion aggregates, as well as spectrally discriminate Aβ and tau aggregates, were investigated. An anionic pentameric LCO was subjected to chemical engineering by: 1) replacing thiophene units with selenophene or phenylene moieties, or 2) alternating the anionic substituents along the thiophene backbone. In addition, two asymmetric tetrameric ligands were generated. Overall, the results from this study identified conformational freedom and extended conjugation of the conjugated backbone as crucial determinants for obtaining superior thiophene-based optical ligands for sensitive detection and spectral assignment of disease-associated protein aggregates.

## Introduction

Accumulation of protein deposits, so-called amyloid, is the histopathological hallmark of several devastating diseases,^1^, ^2^ and the development of sensitive optical ligands to detect and characterize these disease-associated structures is of great interest. In this regard, luminescent conjugated oligo- and polythiophenes (LCOs and LCPs) have been used to study protein aggregates in a variety of recombinant, cellular and tissue-based platforms.^3^–^10^ In addition, chemically defined LCOs are able to pass the blood–brain barrier and selectively stain protein aggregates of amyloid-β (Aβ)^3^ and tau^11^ in transgenic mouse models with Alzheimer’s disease (AD)-like pathology.

The major advantages of thiophene-based probes over conventional amyloid ligands, such as Congo red, thioflavin T (ThT) and their derivatives, are their ability to identify a broader subset of disease-associated protein aggregates and their utilization for spectroscopic assignment of heterogeneous populations of deposits or distinct protein aggregates. The latter property is achieved due to a direct connection between the conformational geometry of the oligothiophene backbone and the emitted light from the dye.^12^, ^13^ This conformation-induced optical read-out has been utilized as an indirect measurement of protein aggregate morphology^3^–^11^ as well as for sensing of other biological processes.^14^–^17^ LCO ligands have proved useful for gaining fundamental novel insights regarding the structural heterogeneity reported for infectious prions as well as Aβ aggregates,^7^, ^8^ and optical identification of protein aggregates with distinct topologies are highly important. For instance, it was recently proposed that the pathogenesis of both AD^18^, ^19^ and prion^20^ diseases might involve two distinct phases, an initial build-up of amyloid followed by a toxic pathway, and that these phases entail different aggregate topologies. Moreover, several lines of evidence indicate that aggregated oligomeric assemblies preceding the formation of mature amyloid fibrils are the culprits of many protein aggregation diseases^21^–^25^ and optical ligands with the capacity to detect these species are therefore highly sought after.

One of the criteria in the definition of amyloid is that the protein aggregate needs to exhibit both affinity for Congo red and green birefringence when viewed by polarization microscopy.^26^ However, it was recently shown that Congo red failed to identify an artificial amyloid structurally indistinguishable from HET-s amyloid^27^ and several studies have demonstrated that Congo red and thioflavins only detect a limited amount of disease-associated prion aggregates,^8^ Aβ assemblies^3^, ^5^ as well as protein inclusion bodies.^28^ These reports underline the importance of identifying and understanding the variety of amyloid topologies that are identified with distinct ligands, and highlight the need for developing versatile amyloid ligands. By a chemical engineering approach, involving the screening of a library of structurally related compounds towards defined amyloid targets, insights regarding the optimal design of such ligands can be achieved. When comparing the optical performance of a library of anionic LCOs, it was recently shown that a backbone consisting of at least five thiophene units with carboxyl groups extending the conjugation length was required to spectrally discriminate Aβ plaques and tau neurofibrillary tangles (NFTs) in AD brain tissue sections.^5^ Moreover, LCOs having at least five thiophene units allowed optical detection of early in vitro formed aggregated species of Aβ, bovine insulin or lysozyme that went undetected with ThT.^4^, ^5^ Hence, the performance of LCOs as amyloid ligands is highly dependent on the length of the thiophene backbone as well as the positioning of anionic side chains along the backbone.

Herein we report the synthesis of a set of novel thiophene-based amyloid ligands designed with minor variations in the chemical structure to restrict or increase the conformational flexibility of the conjugated backbone. The effects of the structural modifications were investigated by comparing the optical performances of these ligands in the detection of a variety of protein aggregates, including in vitro formed aggregated species of Aβ1–42, Aβ deposits and NFTs in AD brain tissue sections, as well as NFTs and non-congophilic prion aggregates in transgenic mice. By replacing distinct thiophene moieties with selenophene or phenylene units and by changing the structural symmetry of the thiophene backbone, further insights regarding the conformation-induced optical performance of this class of amyloid ligands were achieved. The obtained results clearly demonstrate how subtle changes in the chemical composition of the ligand have a large impact on its performance, and the findings might further aid in the understanding of how to design optimal amyloid binding ligands.

## Results and Discussion

**Figure 1:**
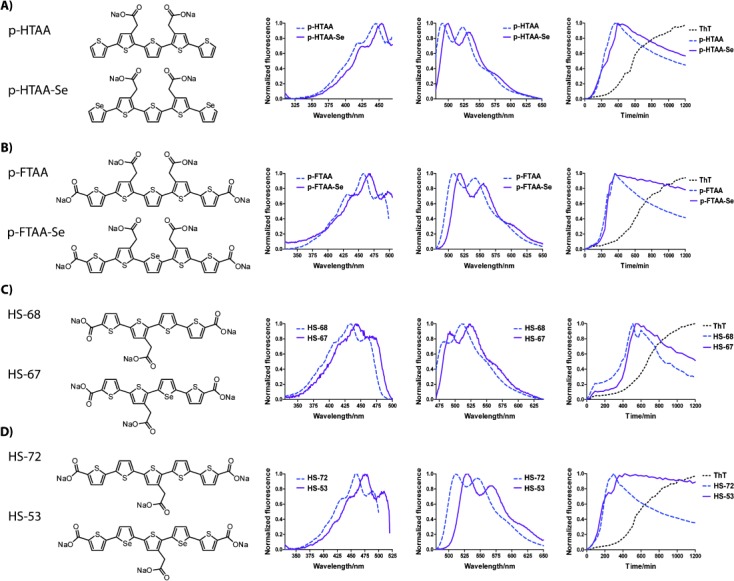
Chemical structures of p-HTAA, p-HTAA-Se, p-FTAA, p-FTAA-Se, HS-68, HS-67, HS-72 and HS-53, and their optical characterization towards in vitro formed recombinant Aβ1–42 fibrils. The first panel displays the chemical structure of the oligothiophene and the corresponding thiophene/selenophene counterpart. The spectral graphs display excitation (second panel) and emission (third panel) spectra of the different ligands in PBS with fibrillar Aβ1–42. All probes show well-resolved excitation and emission spectra when binding to Aβ fibrils. The fourth panel shows the fibrillation of recombinant Aβ1–42 monitored by fluorescence from ThT or the respective ligand. All the pentameric ligands detect early non-thioflavinophilic species in the fibrillation pathway, whereas the tetramers show a high emission at the same time as the onset of ThT.

**Synthesis and optical characterization of LCOs and thiophene/selenophene co-oligomers**: The conformation-induced optical properties of LCO-based amyloid binding ligands have previously been shown to be highly dependent on the length of the thiophene backbone and the positioning of carboxyl moieties along the conjugated backbone.^3^, ^5^ To further investigate the chemical basis for obtaining the conformation-induced optical properties that distinguish LCOs from other conventional amyloid ligands, a novel set of ligands derived from the previously reported LCOs p-FTAA and p-HTAA were synthesized. In total, the library contained two tetramers and six pentamers, including p-FTAA and p-HTAA (Figure 1).

**Scheme 1:**
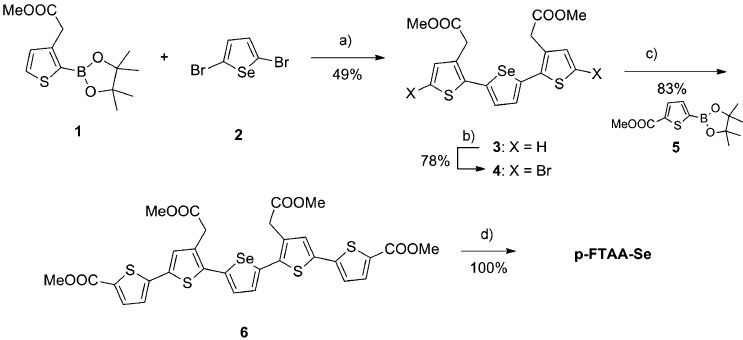
Synthesis of p-FTAA-Se. Reagents and conditions: a) 1,4-dioxane/MeOH, PEPPSI-IPr, K_2_CO_3_, 70 °C, 20 min; b) NBS, DMF, 0 °C to RT, 16 h; c) 1,4-dioxane/MeOH, PEPPSI-IPr, K_2_CO_3_, 70 °C, 20 min; d) NaOH (1 m), 1,4-dioxane, 50 °C, 16 h.

**Scheme 2:**
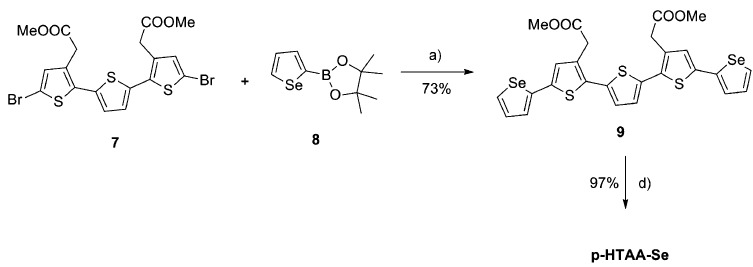
Synthesis of p-HTAA-Se. Reagents and conditions: a) 1,4-dioxane/MeOH, PEPPSI-IPr, K_2_CO_3_, 70 °C, 20 min; b) NBS, DMF, 0 °C to RT, 16 h; c) 1,4-dioxane/MeOH, PEPPSI-IPr, K_2_CO_3_, 70 °C, 20 min; d) NaOH (1 m), 1,4-dioxane, 50 °C, 16 h.

It has been shown, both theoretically and experimentally, that oligoselenophenes have a more quinoid character^29^ and a lower band gap,^30^–^32^ and are more difficult to twist than oligothiophenes.^29^, ^33^ Thus, distinct thiophene units were replaced by a selenophene to render the thiophene/selenophene co-oligomers p-FTAA-Se and p-HTAA-Se (Figure 1). The synthesis of p-FTAA-Se and p-HTAA-Se is depicted in Scheme 1 and Scheme 2, respectively. The preparation of pentamers was carried out by following a similar protocol to that described in our previous work,^3^, ^5^ which takes advantage of Suzuki cross-coupling reactions using PEPPSI-IPr as the source of palladium and potassium carbonate as base (for more details see the Experimental Section in the Supporting Information). Accordingly, the initial efforts were focused on the development of a practical method towards the trimer (**4**) through incorporation of a central selenophene within the desired oligomer. The existing routes to this type of trimer generally involve cross-coupling reactions between 2,5-thiophene diboronic acid/ester and arylbromide compounds. Despite the fact that the synthesis of 2,5-thiophenediylbisboronic acid is convenient,^34^, ^35^ our initial results confirmed that diborylation of selenophene was unfeasible. Therefore, an alternative strategy was used involving the treatment of 2,5-dibromoselenophene (**2**)^36^ with methyl 2-(2-(4,4,5,5-tetramethyl-1,3,2-dioxaborolan-2-yl)thiophen-3-yl)acetate (**1**). The latter compound was prepared by taking advantage of protocols developed by Murata et al. and Christophersen et al.^37^, ^38^ using palladium-catalyzed borylation of pinacolborane and methyl 2-(2-bromothiophen-3-yl)acetate (**12**).^5^ Subsequently, the trimer was dibrominated in the α-positions by using a twofold excess of NBS and then 5-(methoxy carbonyl)thiophene-2-boronic acid, pinacol ester (**5**) was coupled, by using Suzuki chemistry, to give the pentameric thiophene/selenophene co-oligomer **6**. Furthermore, treatment of the trimer **7**^5^ with 4,4,5,5-tetramethyl-2-(selenophen-2-yl)-1,3,2-dioxaborolane (**8**), which was prepared according to a literature procedure,^36^ gave compound **9**, the precursor to p-HTAA-Se. The two pentamers were hydrolyzed by using sodium hydroxide in dioxane/water (1.5 equiv NaOH/ester) to give the target compounds, p-FTAA-Se and p-HTAA-Se, as the sodium salts.

**Scheme 3:**
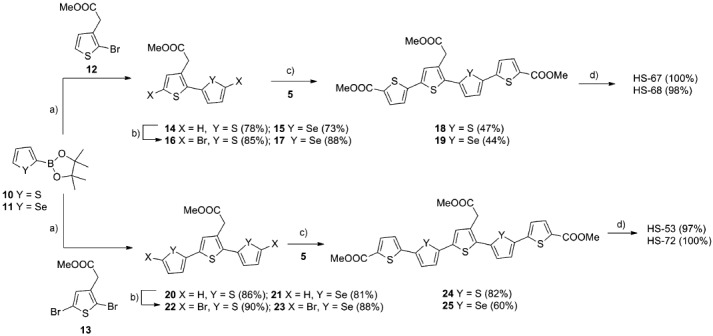
Synthesis of HS-67, HS-68, HS-53 and HS-72. Reagents and conditions: a) 1,4-dioxane/MeOH, PEPPSI-IPr, K_2_CO_3_, 70 °C, 20 min; b) NBS, DMF, 0 °C to RT, 16 h; c) 1,4-dioxane/MeOH, PEPPSI-IPr, K_2_CO_3_, 70 °C, 20 min; d) NaOH (1 m), 1,4-dioxane, 50 °C, 16 h.

It is generally known that side chain substitution on neighboring thiophene rings causes oligothiophenes to become nonplanar, and so far almost all of the previously reported LCOs have been synthesized from a trimer building block, having 3-thiophene acetic acid attached to the 2- and 5-position of an unsubstituted thiophene.^3^, ^5^ To increase the conformational variability in the LCO library, a set of asymmetrical tetrameric and pentameric LCOs having only one 3-thiophene acetic acid unit along the backbone were generated (Figure 1, Scheme 3). In addition, similar to as described above, distinct thiophene units were replaced by selenophene to render the thiophene/selenophene co-oligomer analogue (Scheme 3). Treatment of commercially available thiophene-2-boronic acid, pinacol ester **10** and the corresponding selenophene-2-boronic acid, pinacol ester **11**^35^ with the monomer **12** and **13** followed by bromination gave the dimers **16** and **17** or trimers **22** and **23**. These intermediates were subsequently coupled with 5-(methoxy carbonyl)thiophene-2-boronic acid, pinacol ester (**5**) to obtain target tetramers **18** and **19** or pentamers **24** and **25**. The final products were subsequently transformed to salt form by hydrolysis with NaOH to provide HS-68, HS-67, HS-72 and HS-53.

When diluted in phosphate buffered saline (PBS, pH 7.4) all included co-oligomers displayed bathochromic shifts of excitation and emission spectra as compared with the corresponding oligothiophene (Supporting Information, Figure S1). The most pronounced red shift in both excitation and emission mode was accomplished when alternating two selenophenes and three thiophenes in an asymmetric pentameric backbone (HS-53, Supporting Information, Figure S1 D), whereas peripheral placement of the selenophenes (p-HTAA-Se, Supporting Information, Figure S1 A) exerted the smallest effect on the optical behavior. Earlier studies have confirmed that an increase in the number of selenophene units in pentameric co-oligomers of thiophene and selenophene results in a bathochromic shift, as the selenium heteroatom lowers the band gap by stabilizing the LUMO level.^30^, ^31^, ^36^ In addition, the red shift might also be observed due to an increased quinoid character of the selenophene giving the inter-ring C–C bond more double-bond properties and an enhanced efficiency of conjugation through the backbone.^29^ In comparison with their thiophene analogues, the selenophene-containing ligands also showed a decrease in the fluorescence intensity. The phenomenon has been reported previously in a study with thiophene and selenophene co-polymers^39^ and might be dependent on the lowering of the band gap increasing the probability of nonradiative emission.

**Characterization of in vitro formed recombinant Aβ1**–**42 fibrils**: In the next series of experiments, LCOs and thiophene/selenophene co-oligomers were tested on recombinant Aβ1–42 fibrils. Overall, intercalation with the amyloid fibrils resulted in enhanced fluorescence and well-resolved excitation and emission spectra for both LCOs and co-oligomers; hence, all probes showed specific binding to Aβ fibrils and the resulting conformational restriction of the backbones was mirrored in the defined spectra (Figure 1). In comparison with probe diluted in PBS, each probe consistently showed a red shift in excitation spectrum when mixed with Aβ fibrils. Additionally, intercalation with the fibrils caused a blue shift in the emission spectrum for all probes. Similar to the result in PBS, introducing selenophenes in the thiophene backbone caused a shift in excitation and emission spectra towards longer wavelengths when the probes were mixed with Aβ fibrils, and the largest bathochromic shifts in both modes were seen with HS-53 (Figure 1 D). As mentioned above, HS-53 showed the most pronounced shifts also in PBS indicating that the design with alternating thiophenes and selenophenes in the backbone has the largest effect on the resulting optical properties when compared to the thiophene analogue. The tetrameric and pentameric ligands were then utilized to monitor the kinetics of recombinant Aβ1–42 fibril formation. The fibrillation was performed under physiological conditions (37 °C, pH 7.4, quiescent), a protocol that has previously been reported to result in the typical sigmoidal curve of amyloid formation when ThT fluorescence is assessed as read-out for the reaction.^5^ The fluorescence from each probe and the reference ThT was used to continuously monitor the fibrillation kinetics, and the emission intensities were plotted against time (Figure 1). In accordance with earlier observations, all pentameric ligands displayed an Aβ1–42 kinetic graph with a short lag time followed by a steep growth phase and then a decreasing plateau. The replacement of thiophenes with selenophenes did not have any significant effect, as the resulting graphs for pentameric LCOs and corresponding co-oligomers were more or less identical. All included LCOs and co-oligomers with a pentameric backbone showed an onset of fluorescence more than 200 min earlier than ThT. Previous studies using transmission electron microscopy have shown the presence of small fibrillar species during the earlier time-points with observed strong p-FTAA emission, whereas the onset of ThT fluorescence and decreased p-FTAA emission was assigned to the presence of larger bundles of amyloid fibrils.^3^, ^5^ Hence, in agreement with earlier findings, all pentameric thiophene- and selenophene-based ligands displayed a strong emission when bound to non-thioflavinophilic species preceding mature Aβ fibrils. Introducing selenophenes or asymmetry along the pentameric backbone did not affect the optical performance of LCOs for detection of early in vitro formed aggregated species of Aβ.

The asymmetric tetramers HS-67 and HS-68 displayed an Aβ1–42 kinetic graph with a short lag time followed by a temporary increase in the fluorescence at the same time point as the fluorescence onset of the pentameric ligands, and then a second continuous growth phase corresponding to the onset of ThT emission. Hence, HS-67 and HS-68 seem to interact with aggregated non-thioflavinophilic species, observed as a slight increase in emission, but as with ThT, the tetramers need bundles of fibrils in order to convert into a highly emissive state. A similar Aβ1–42 amyloid-formation curve, but with a slightly less pronounced initial growth phase, was observed for q-FTAA, an asymmetrical tetramer substituted with a carboxyl group at one of the end α-positions, whereas with the tetramers t-HTAA and q-HTAA, which lack this carboxyl group, the early increase in fluorescence was almost absent.^5^ Hence, extending the conjugated backbone with carboxyl groups increases the emission from tetrameric dyes bound to aggregated non-thioflavinophilic Aβ1–42 species. However, when the excitation mode was utilized to monitor the fibrillation kinetics, it was clearly shown that the conformation-induced highly emissive state for HS-68 was obtained at a similar time point as for ThT, whereas p-FTAA and p-HTAA displayed an earlier conformation-induced onset (Supporting Information, Figure S2). Overall, in agreement with previous studies,^3^–^5^ we conclude that a conjugated backbone consisting of at least five units was necessary to achieve an amyloid ligand displaying strong emission upon binding to aggregated nonthioflavinophilic Aβ1–42 species.

**Figure 2:**
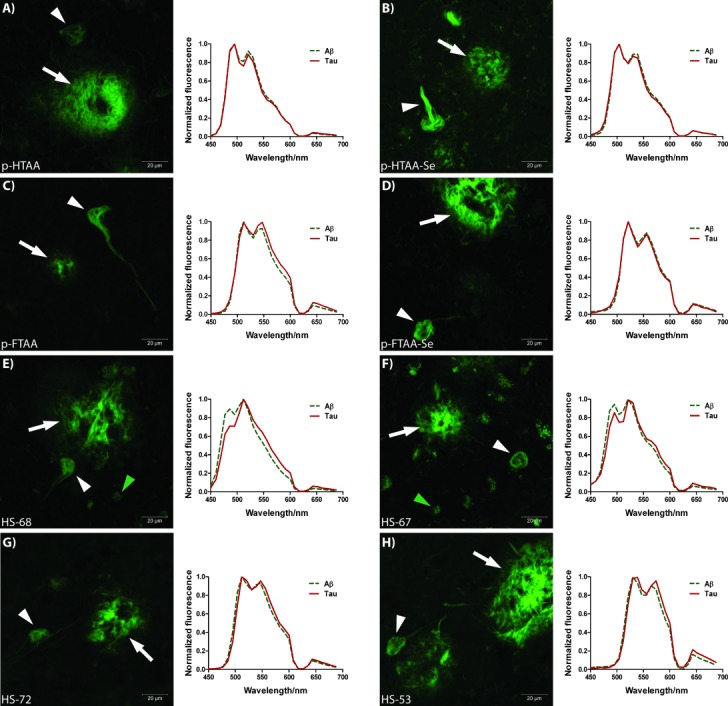
Fluorescence images and emission spectra of p-HTAA, p-HAA-Se, p-FTAA, p-FTAA-Se, HS-68, HS-67, HS-72 and HS-53 bound to two histopathological hallmarks of AD (Aβ plaques and NFTs) in human brain tissue. The emission spectra of the indicated ligand bound to Aβ plaques (green dashed line) or NFTs (red solid line) when excited at 405 nm. The images show the typical pathological protein entities, Aβ plaques (white arrows) and NFTs (white arrow heads), from which the emission spectra were obtained. Green arrowheads in E and F indicate lipofuscin. Scale bars represent 20 μm.

**Characterization of Aβ plaques and neurofibrillary tangles in brain tissue sections**: Previous studies have shown that pentameric and heptameric LCOs identify a broader subset of Aβ deposits in brain tissue sections than conventional amyloid ligands.^3^, ^5^, ^40^ The staining pattern of pentameric and heptameric LCOs resembled the overall Aβ burden obtained with Aβ immunohistochemistry.^3^, ^40^ In addition, an LCO consisting of at least five thiophene units with carboxyl groups extending the conjugation length was required to spectrally discriminate Aβ aggregates and tau NFTs, the two major histopathological hallmarks of AD, in AD brain tissue sections.^3^, ^5^ To gain further insight regarding the underlying molecular requirements for achieving an LCO-based ligand for spectral separation of Aβ aggregates and NFTs, the library of ligands was applied to frozen AD brain sections. All included LCOs and thiophene/selenophene co-oligomers showed specific binding to Aβ aggregates and NFTs (Figure 2). When excited at 405 nm, all included probes showed well-resolved emission spectra with characteristic double peaks upon binding to assemblies of Aβ and tau, denoting conformational restrictions of the backbone (Figure 2). Similar with the spectral results obtained in buffer and with recombinant Aβ fibrils, the thiophene/selenophene co-oligomers displayed a red shift in emission compared to their thiophene analogues owing to the selenium containing unit(s) (Figure 2).

**Figure 3:**
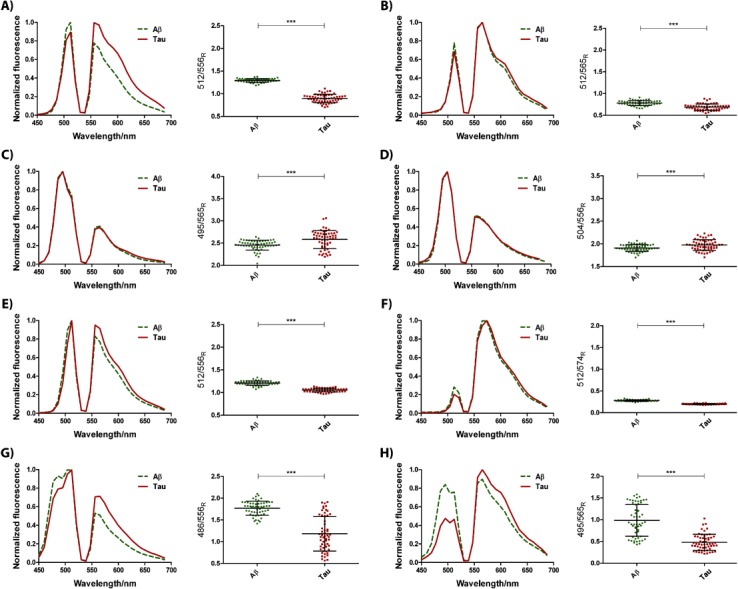
Emission spectra and ratio plot for: A) p-FTAA, B) p-FTAA-Se, C) p-HTAA, D) p-HTAA-Se, E) HS-72, F) HS-53, G) HS-68, and H) HS-67 bound to pathological hallmarks in AD human brain tissue. The emission spectra of the respective ligand bound to Aβ plaques (green dashed line) or NFTs (red solid line), when the ligand has been excited with two distinct wavelengths and the resulting emission spectra have been merged together. Plot of the ratio of light intensity emitted at the indicated wavelengths for each probe when bound to Aβ plaques (green triangles) or NFTs (red squares) shown with standard deviation. The intensities are obtained from the merged emission spectra.

Clearly, introducing selenophenes or asymmetry to the molecule did not impair the amyloid-binding properties; however, spectral analysis of the ligands bound to Aβ aggregates or NFTs revealed that the structural modifications had major impact on the ability for spectral separation of Aβ plaques and NFTs. In previous studies,^3^, ^5^ p-FTAA has emerged as the optimal spectral separator of Aβ plaques and NFTs in AD brain tissue. Herein, when p-FTAA was excited at a single wavelength (405 nm), the emission signature of both Aβ and tau peaked at identical wavelengths (Figure 2 C), however, the higher value of the last tau peak as well as the more pronounced shoulder at 590 nm confirmed the earlier reported red shift of p-FTAA bound to NFTs in comparison with Aβ. The difference in emission was further assessed by simultaneously exciting the probe at 405 and 538 nm, an operation that resulted in one single emission spectrum in which both green and red contributions in emission were highlighted (Figure 3 A). By plotting the ratio of the fluorescence intensity observed for the separated peaks, the dissimilarity of the obtained spectral fingerprints became obvious, and the shift of p-FTAA upon binding to misfolded tau was clearly illustrated and shown to be significantly different with a difference between the means of −0.396±0.013 (Figure 3 A, and Supporting Information, Table S1). Interestingly, the spectral analysis of AD brain tissue stained with p-FTAA-Se revealed almost identical emission spectra for the two protein entities and the gap between the mean values (difference between means=−0.094±0.012) in the ratio plot was considerably decreased, although still significantly different (Figure 3 B, and Supporting Information, Table S1). In addition, the *R*^2^ value from the unpaired t-test was 0.8901 for p-FTAA meaning that 89 % of all the variation among the obtained values could be ascribed to differences between the two group means, whereas 11 % of the variation resulted from scatter among the values within the groups. The same number for p-FTAA-Se was considerably lower (*R*^2^=0.3421). Hence, by replacing the central thiophene unit with a selenophene, the distinct spectral separation of Aβ and tau achieved with p-FTAA was substantially reduced. As earlier studies have shown that oligoselenophenes are more difficult to twist than oligothiophenes,^29^, ^33^ the introduction of a selenophene unit between the two 3-thiophene acetic moieties might restrict the conformational flexibility of the molecule. Ongoing studies with solid state NMR spectroscopy and molecular modeling (work in progress) have indeed shown that these groups, in combination with the carboxyl groups at the α-end positions, are important for achieving an optimal interaction between the LCO and the amyloid. Similar to what was recently reported for Congo red,^27^ the LCOs seem to preferably intercalate in a groove along the amyloid fibril axis and the carboxyl moieties interact with cationic residues flanking this groove. As previously reported, replacing the end carboxyl moiety with hydrogen, resulting in p-HTAA or p-HTAA-Se, also diminished the spectral separation of Aβ plaques and NFTs (Figure 3 C and D). The emission spectra and ratio plot for p-HTAA demonstrated a small shift of tau against shorter wavelengths, a phenomenon that was observed earlier for the hexameric probe hx-HTAA.^5^ The same tendency, although to a smaller degree, was also seen in the emission spectra for the selenophene-containing counterpart, p-HTAA-Se. The statistical values from the unpaired t-test were also considerably lower (Supporting Information, Table S1) than for the corresponding analogues having end carboxyl moieties. Previous studies^3^, ^5^ have not reported p-HTAA to be green shifted upon binding to tau, and this might be explained with the fact that these earlier studies were not performed with a combination of excitation wavelengths or a confocal microscope reducing the light scattering.

The spectral analysis of the ligands with the second modification of the pentameric scaffold, keeping the end carboxyl moieties but having only one 3-thiophene acetic acid moiety in the backbone, verified the necessity of the terminal carboxyl groups for efficient spectral separation of Aβ aggregates and NFTs. When binding to Aβ plaques and NFTs, the asymmetric LCO HS-72 emitted light with a variation in color, although the red shift of tau was not as evident as with p-FTAA (difference between means=−0.1535±0.008382; *R*^2^=0.7529; Figure 3 E, and Supporting Information, Table S1). Similar to the observation for pentameric ligands with a symmetric backbone, introducing selenophenes at distinct positions reduced the spectral shift between Aβ aggregates and NFTs (difference between means=−0.07902±0.002721; Figure 3 F). In addition, the ratio plot of the resulting co-pentamer HS-53 demonstrated a very narrow distribution of the data points and 88 % (*R*^2^=0.8846) of all the variation among values was explained by differences between the two group means (Supporting Information, Table S1). By alternating selenophenes with thiophenes, the rigidity of the conjugated backbone should increase considerably and this conformational restriction might be reflected as a decrease in the spectral difference between Aβ aggregates and NFTs as well as a limited spectral variation for individual aggregated entities.

The most distinct spectral separation of Aβ plaques and NFTs was obtained with the asymmetric tetramers (Figure 3 G and H, and Supporting Information, Table S1). When the tetrameric LCO HS-68 was excited at 405 nm, the first peak of the tau emission spectrum was barely noticeable, confirming that the optical signature of the probe upon binding to the protein was considerably shifted towards longer wavelengths (Figure 2 E). The double excitation protocol and the resulting ratio plot verified the observation and revealed that HS-68 displayed a large spectral variation, especially within the population of misfolded tau. Analogous to the asymmetric pentamers, the effect on the spectral properties of replacing one thiophene with a selenophene was smaller than what was observed with p-FTAA; however, the co-oligomer HS-67 showed a wide spectral distribution for Aβ aggregates instead of NFTs (Figure 3 H, and Supporting Information, Table S1). These distinct variations in emission spectra, both between the two protein entities and within the same protein, are in strong contrast to the earlier results obtained with the tetramers t-HTAA, q-HTAA and q-FTAA, as these dyes did not display any significant spectral distinction between Aβ and tau deposits.^5^ Chemically, HS-68 and q-FTAA contain the identical number of thiophene units and charged groups. The only difference is the positioning of the carboxyl groups along the tetrameric backbone. Firstly, HS-68 has carboxyl groups at both end α-positions, whereas only one of these positions is functionalized with a carboxyl group in q-FTAA, and the importance of these substitutions has been demonstrated previously. It has been suggested that the red shift of tau is caused by the carboxyl end-groups interacting with chemical entities, such as cationic groups, of the protein, and earlier results have shown that the pentameric LCO p-FTAA surpasses both hexameric and heptameric analogues,^5^ showing that the maximum effect of the carboxyl extensions on the electronic system is obtained with a decreasing length of the backbone. The result obtained herein for HS-68 verifies these findings, as this tetrameric dye displayed a pronounced spectral separation of Aβ aggregates and NFTs compared to p-FTAA and HS-72. Secondly, HS-68 has only one acetic acid moiety, whereas q-FTAA is synthesized from a trimer building block having an unsubstituted thiophene ring between two 3-thiophene acetic acid moieties. In contrast to the carboxyl end-groups, an interaction between the acetic acid derived carboxyl moieties with a chemical entity within the amyloid binding groove will lock the acetic acid substituted thiophene rings in rather distinct positions. Hence, upon binding to protein aggregates, the q-FTAA backbone might be more conformationally restricted compared to the HS-68 backbone, and this would, together with the diminution of the shift between Aβ aggregates and NFTs when introducing selenophenes, prove the assumption that the spectral phenomenon is highly dependent on the degree of conformational freedom of the conjugated backbone.

The chemical design of HS-68 and p-FTAA appears to be optimal when it comes to separate Aβ plaques and NFTs in AD. However, it should be noted that the specific correlations between chemical design and spectral separation discussed above should only be applied to the Aβ/tau model system. For example, the most distinct spectral discrimination of prion aggregates associated with distinct prion strains has been achieved with polythiophene acetic acid (PTAA), having acetic acid substitution on all thiophene units.^8^

**Figure 4:**
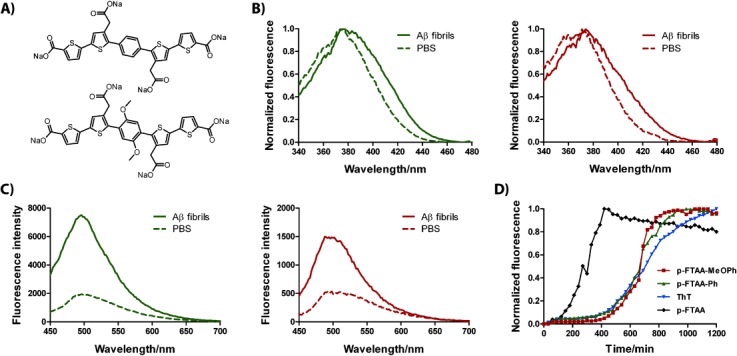
A) The chemical structure of p-FTAA-Ph (top) and p-FTAA-MeOPh (bottom). B) Excitation, and C) emission spectra of p-FTAA-Ph (green) and p-FTAA-MeOPh (red) in PBS (dashed line) or mixed with in vitro formed recombinant Aβ1–42 fibrils (solid line). The excitation/emission wavelengths were 410/500 nm. D) The fibrillation of recombinant Aβ1–42 monitored by fluorescence from ThT, p-FTAA, p-FTAA-Ph or p-FTAA-MeOPh. p-FTAA detects early non-thioflavinophilic species in the fibrillation pathway, whereas p-FTAA-Ph and p-FTAA-MeOPh showed a high emission at the same time as the onset of ThT.

**Scheme 4:**
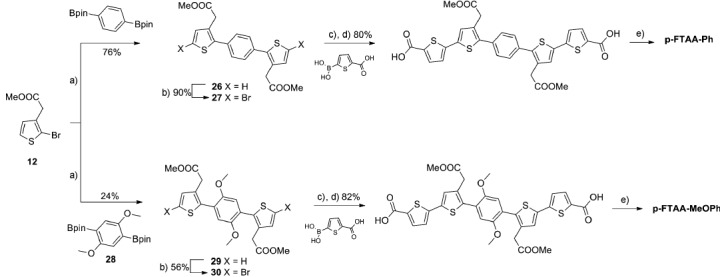
Synthesis of p-FTAA-Ph and p-FTAA-MeOPh. Reagents and conditions: a) 1,4-dioxane/MeOH, PEPPSI-IPr, K_2_CO_3_, 70 °C, 20 min; b) NBS, DMF, 0 °C to RT, 16 h; c) 1,4-dioxane/MeOH, PEPPSITM-IPr, K_2_CO_3_, 70 °C, 20 min; d) NaOH (1 m), 1,4-dioxane, 50 °C, 16 h.

**Synthesis and characterization of thiophene/phenylene co-oligomers**: Overall, the results described above further proved the previously shown correlation between LCO structure and amyloid spectral fingerprinting. When monitoring the kinetics of Aβ1–42, the length of the conjugated backbone had to exceed four units to strongly fluoresce upon binding to early formed aggregates not reactive with ThT. In addition, from the histology experiments, the level of conformational freedom along the conjugated backbone was identified as a novel criterion for spectral separation of Aβ aggregates and NFTs. To verify all these findings, p-FTAA was subjected to further molecular engineering and as phenyl moieties previously have been utilized to disrupt the planarity of oligothiophenes,^41^–^43^ two co-oligomers termed p-FTAA-Ph and p-FTAA-MeOPh with the central thiophene ring replaced with a phenyl ring, were synthesized (Figure 4, Scheme 4). The aim with these designs was to create pentameric analogues to p-FTAA that have a nonplanar backbone with a disrupted conjugation length as well as a sterically restricted center.

The preparation of p-FTAA-Ph and p-FTAA-MeOPh was carried out following the similar concept described above, utilizing Suzuki cross-coupling reactions (Scheme 4). In PBS, both the ligands displayed blue-shifted excitation spectra compared to p-FTAA (Figure 4 B, and Supporting Information, Figure S1 B), and when mixed with recombinant Aβ1–42 fibrils, the excitation spectra were only slightly shifted towards longer wavelengths. Hence, the introduction of a central phenyl ring diminished the well-resolved excitation spectrum observed for all the other pentameric ligands interacting with recombinant Aβ1–42 fibrils. This observation was also reflected in the emission spectra, as the interaction with the amyloid fibrils resulted only in enhanced fluorescence from p-FTAA-Ph and p-FTAA-MeOPh. In the Aβ1–42 fibrillation experiment, p-FTAA-Ph and p-FTAA-MeOPh displayed an extended lag phase compared to p-FTAA, and the kinetic graphs almost completely overlapped with the one obtained with ThT (Figure 4 D). Hence, by replacing the central thiophene ring with a phenyl ring, the ability to detect early formed non-thioflavinophilic aggregates of Aβ1–42 was abolished and amyloid ligand properties more similar to ThT than pentameric thiophene-based molecules were acquired.

**Figure 5:**
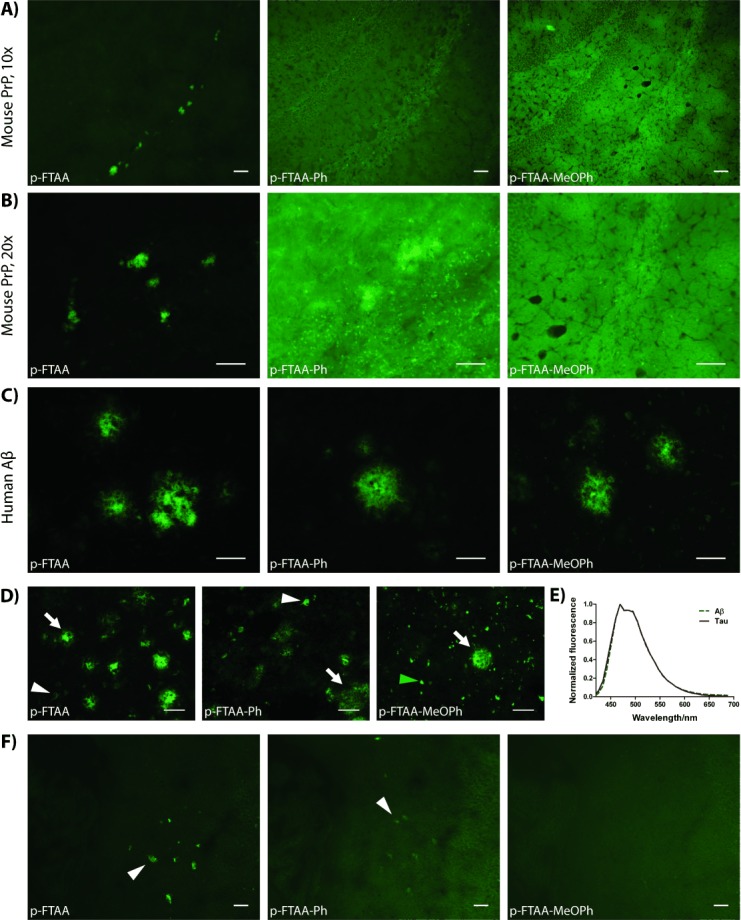
Fluorescence images of: A), B) prion infected transgenic mice, C), D) AD, or F) tau^P301S^ transgenic mice brain sections stained with p-FTAA, p-FTAA-Ph or p-FTAA-MeOPh. White arrows and arrowheads indicate Aβ plaques and NFTs, respectively, whereas the green arrowhead identifies lipofuscin. Scale bars represent 50 μm. E) Emission spectra collected from Aβ plaques (green dashed line) and tau tangles (red solid line) labeled with p-FTAA-Ph.

To further explore the findings from the in vitro fibrillation experiments, p-FTAA-Ph and p-FTAA-MeOPh was next applied to tissue sections from AD brain as well as prion infected transgenic mice with prion aggregates that were identified by oligo- and polythiophenes but went undetected by ThT and Congo red.^44^ As shown in Figure 5, p-FTAA emission could easily be utilized to detect the prion aggregates and the resulting strong fluorescence allowed detection of also very small-sized deposits. These prion aggregates were also detected by all the other oligothiophenes or thiophene/selenophene co-oligomers (Supporting Information, Figure S3). With p-FTAA-Ph, it was very difficult to distinguish aggregates from the surrounding tissue and the exposure time used in the fluorescence image was more than 60-times longer than for p-FTAA (Figure 5). With p-FTAA-MeOPh, we could not identify any prion aggregates, indicating that this dye performs similar to ThT and Congo red. To rule out the possibility that the marked difference in fluorescence intensity was an inherent property of the probes, their labeling of congophilic Aβ plaques in AD brain tissue was compared (Figure 5 C). p-FTAA-Ph showed weaker fluorescence also from Aβ deposits, but the exposure time was only three-times longer than the one used for p-FTAA. In addition, congophilic Aβ plaques could be readily identified with p-FTAA-MeOPh. Hence, the low intensity from p-FTAA-Ph upon binding to the prion deposits and the lack of binding of p-FTAA-MeOPh to the same aggregated species were only observed for deposits that are not recognized by conventional amyloid ligands. Taken together with the previous findings for the thiophene/selenophene co-oligomers, these observations indicate that an extended conjugation of the backbone is necessary to obtain strong fluorescence signals from non-congophilic and non-thioflavinophilic protein aggregates. The presumed disordered structure found in early in vitro formed Aβ aggregates or in the prion deposits may allow the thiophene/phenylene co-pentamers to bind, but prevents them from adopting a fully emissive conformation. Alternatively, pentameric thiophene/selenophene co-oligomers might identify a wider range of aggregate topologies than p-FTAA-Ph and p-FTAA-MeOPh. In either case, we conclude that replacing the center thiophene ring with a phenyl moiety will diminish sensitive fluorescence detection of a variety of early formed aggregated species or disease-associated protein aggregates.

Next we investigated whether the introduced phenyl moiety in the p-FTAA scaffold had an effect on spectral fingerprinting of Aβ and tau deposits. Both Aβ and tau aggregates were easily identified with p-FTAA-Ph (Figure 5 D). However, the emission spectra from p-FTAA-Ph demonstrated a maximum peak at 469 nm and a pronounced shoulder at 495 nm for both these aggregated species (Figure 5 E). Hence, the distinct spectral separation of Aβ and tau obtained with p-FTAA was completely abolished, although p-FTAA-Ph has carboxyl groups extending the conjugated backbone. As a similar effect, although not as pronounced, was seen when increasing the rigidity of the main chain by replacing the central thiophene with selenophene (Figure 3), it could be concluded that conformational freedom along the conjugated backbone in combination with end carboxyl moieties is necessary for achieving superior spectral separation of the two pathological hallmarks observed in AD. Frozen AD brain sections were also stained by p-FTAA-MeOPh, and as mentioned earlier, Aβ deposits were easily identified due to strong emission from the probe (Figure 5 C and D). However, aggregated tau species, NFTs and dystrophic neurites, were very difficult to detect. In addition, increased background fluorescence, most likely from lipofuscin, was also observed. To verify the almost complete lack of aggregated tau staining from p-FTAA-MeOPh, brain sections from transgenic mice expressing human P301S tau protein^45^ were stained with p-FTAA and its phenyl analogues (Figure 5 F). Analogous to the results obtained for the non-thioflavinophilic and non-congophilic prion aggregates, intracellular tau aggregates in the brain stem could easily be identified by p-FTAA fluorescence, whereas p-FTAA-Ph stained aggregates displayed only weak fluorescence and p-FTAA-MeOPh showed an almost complete lack of aggregated tau staining (Figure 5 F). These observations strengthen our conclusions that substitution of the central thiophene ring with a phenyl moiety will diminish sensitive fluorescence detection of distinct protein aggregates. Furthermore, introducing methoxy substituents on the phenyl ring will completely abolish fluorescence detection of certain protein deposits. Interestingly, a similar methoxy modification on the central phenyl ring of the Congo red analogue, X-34,^46^ has rendered an amyloid ligand, MeO-X-04,^47^ that allows fluorescence detection of Aβ and tau deposits in postmortem sections of AD brain with good specificity. This is in contrast to our finding for conformationally flexible amphiphilic thiophene-based ligands. Recently, it was also reported that amphiphilic *N’*-benzylidene-benzohydrazide (NBB)-based fluorescent amyloid ligands with bulky hydrophilic groups showed selectivity towards tau aggregates, whereas non-amphiphilic NBB ligands were rather selective for Aβ deposits.^48^ Similar to our thiophene ligands, the NBBs display a high degree of rotational freedom, suggesting that introduction of hydrophobic moieties, such as methoxy groups, in amyloid ligands with a flexible molecular scaffold will abolish fluorescence detection of tau aggregates. Whether this phenomenon is associated with lack of binding, inability of the molecule to adopt a highly emissive conformation or a combination of these factors needs to be investigated further. Overall, we conclude that the central thiophene ring in pentameric LCOs is an essential chemical element for sensitive fluorescent assignment of a variety of disease-associated protein aggregates.

## Conclusion

Understanding the optical performance of fluorescent amyloid ligands is crucial for obtaining molecular probes that identify a variety of disease-associated protein aggregates. Herein, we have verified and identified distinct molecular requirements that are highly important for achieving thiophene-based ligands that are superior, compared to conventional ligands, for optical assignment of disease-associated protein aggregates. In addition, we have shown that small chemical modifications of the molecular scaffold significantly influence the diversity of amyloid topologies that are detected or spectrally assigned by the ligand (Table 1). Firstly, replacing the central thiophene ring with a phenyl moiety abolished fluorescent detection of non-thioflavinophilic Aβ species and non-congophilic prion aggregates. Secondly, the level of conformational freedom along the conjugated backbone was identified as a novel criterion for spectral separation of Aβ aggregates and NFTs, the two major pathological hallmarks of AD. Overall, we have demonstrated that a combination of fluorescent ligands will facilitate the detection of a broader subset of amyloid topologies, and the results presented also underline the importance of understanding the variety of disease-associated protein aggregates that are identified with distinct ligands.

**Table 1:** Amyloid topologies detected and spectrally assigned by the respective ligand.

Probe	Chemical structure	Detection of ThT negative Aβ aggregates	Spectral separation of Aβ and tau	Detection of ThT negative prion aggregates
p-FTAA	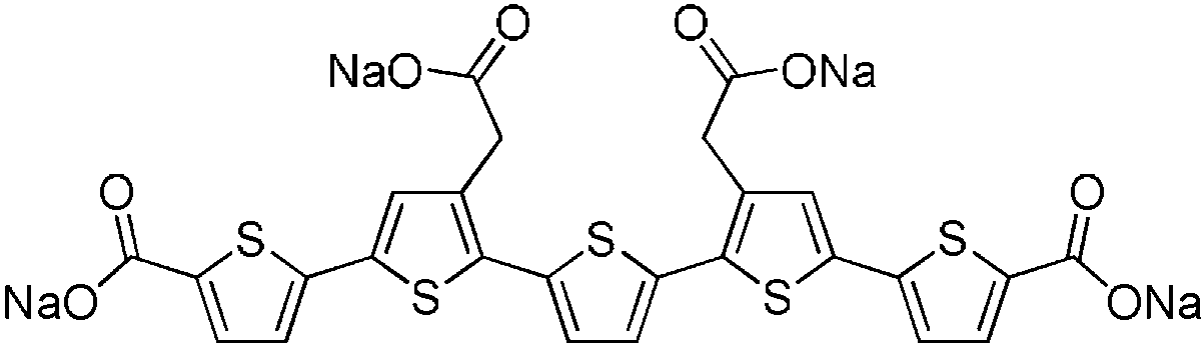	+++	+++	+++
p-FTAA-Se	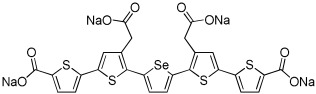	+++	+	++
p-FTAA-Ph	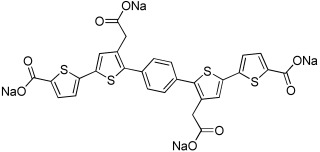	−	−	−
p-FTAA-MeOPh	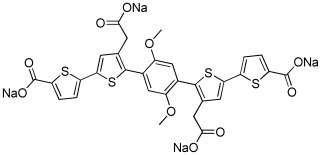	−	−	−
p-HTAA	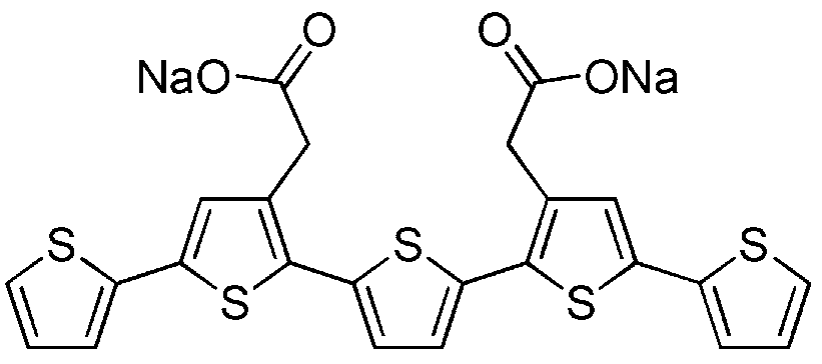	+++	+	+++
p-HTAA-Se	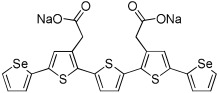	+++	+	++
HS-72	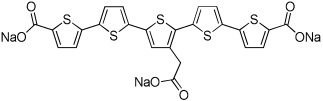	+++	++	++
HS-53	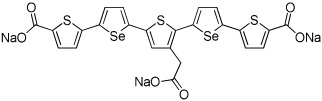	+++	+	++
HS-68	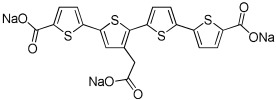	−	+++	+
HS-67	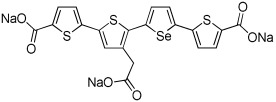	−	+++	+

## Experimental Section

Full experimental details including additional characterization data and NMR spectra of new compounds are given in the Supporting Information.
