# Supplementary material for: The Structural Basis for Optimal Performance of Oligothiophene-Based Fluorescent Amyloid Ligands: Conformational Flexibility is Essential for Spectral Assignment of a Diversity of Protein Aggregates
Source: Chemistry. 2013 Jun 18;19(31):10179–92. doi: 10.1002/chem.201301463 (PMC3884759; doi:10.1002/chem.201301463)
Supplement: Supplementary file 1 [file chem0019-10179-sd1.pdf]

CHEMISTRY
A EUROPEAN JOURNAL

Supporting Information

© Copyright Wiley-VCH Verlag GmbH & Co. KGaA, 69451 Weinheim, 2013

The Structural Basis for Optimal Performance of Oligothiophene-Based Fluorescent Amyloid Ligands: Conformational Flexibility is Essential for Spectral Assignment of a Diversity of Protein Aggregates

Therése Klingstedt,[a] Hamid Shirani,[a] K. O. Andreas Åslund,[a] Nigel J. Cairns,[b] Christina J. Sigurdson,[c] Michel Goedert,[d] and K. Peter R. Nilsson*[a]

chem_201301463_sm_miscellaneous_information.pdf

Supporting information

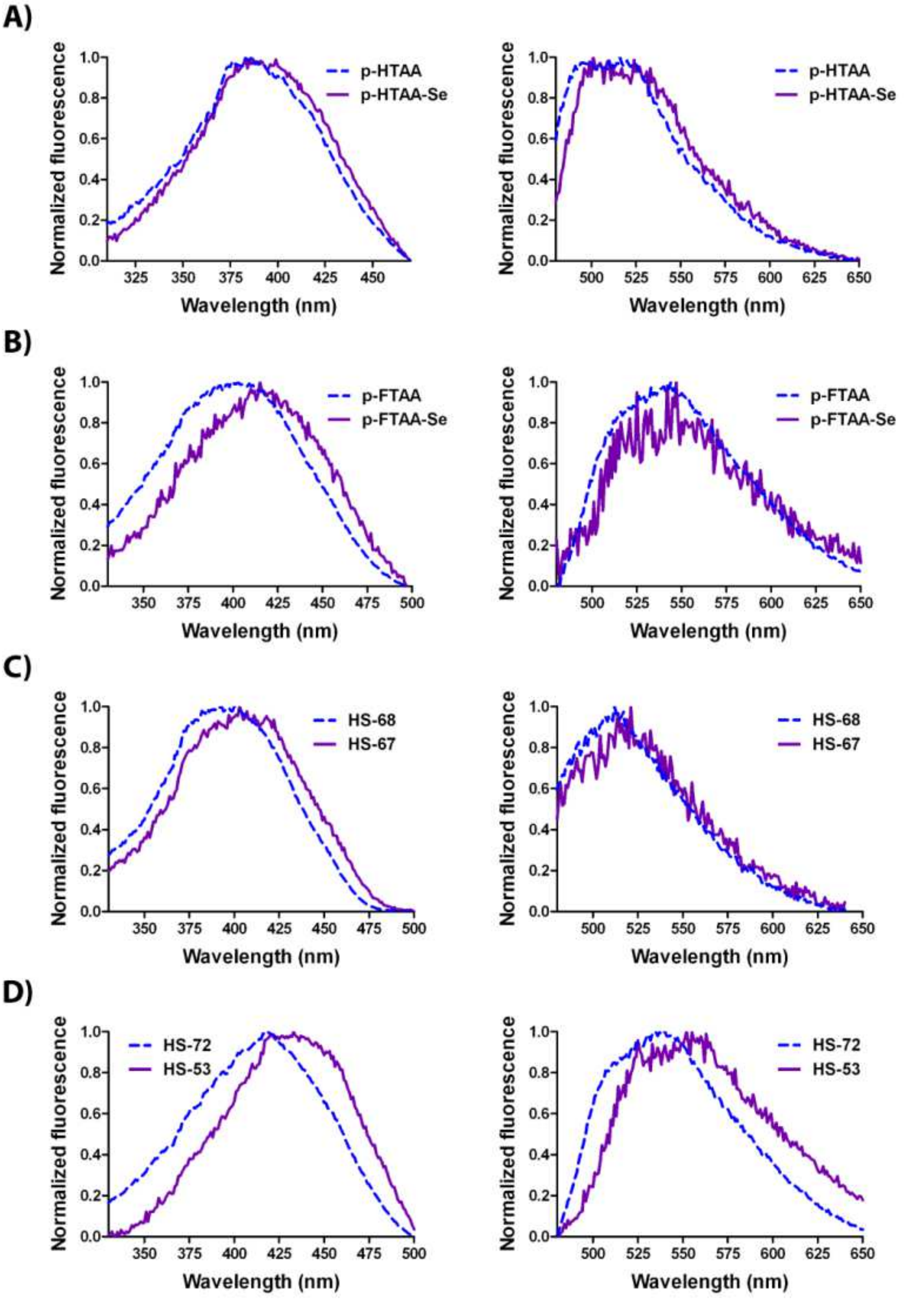

Figure S1: Excitation (left panel) and emission (right panel) spectra of 300 nM LCOs or their corresponding thiophene/selenophene co-oligomer dissolved in PBS pH 7.4. A) p-HTAA (blue) and p-HTAA-Se (magenta). B) p-FTAA (blue) and p-FTAA-Se (magenta). C) HS-68 (blue) and HS-67 (magenta). D) HS-72 (blue) and HS-53 (magenta).

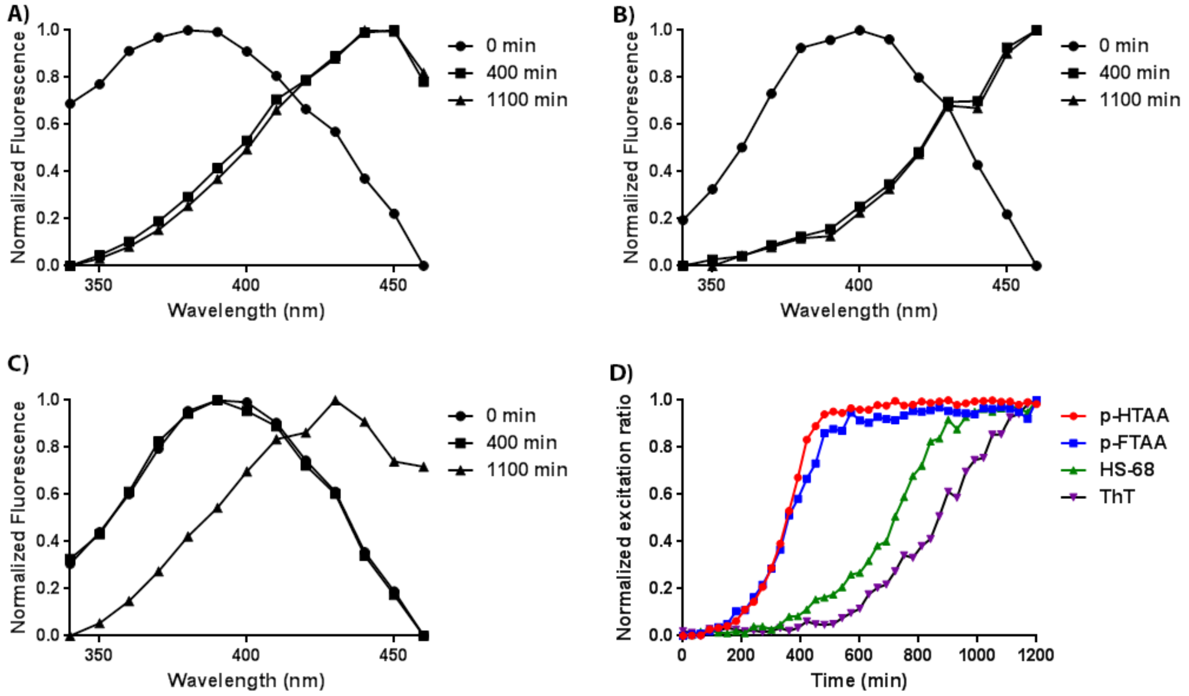

Figure S2: Excitation spectra of p-HTAA (A), p-FTAA (B) and HS-68 (C) after 0 min, 400 min and 1100 min of Aβ1-42 fibrillation. The emission wavelength was fixed at 520 nm. D) The fibrillation of recombinant Aβ1-42 monitored by normalized excitation ratio from p-HTAA (Ratio 450/380nm), p-FTAA (Ratio 450/400nm), HS-68 (Ratio 440/390nm) or ThT (Ratio 440/360nm). p-HTAA and p-FTAA detect early non-thioflavinophilic species in the fibrillation pathway, whereas HS-68 displayed a similar onset as ThT.

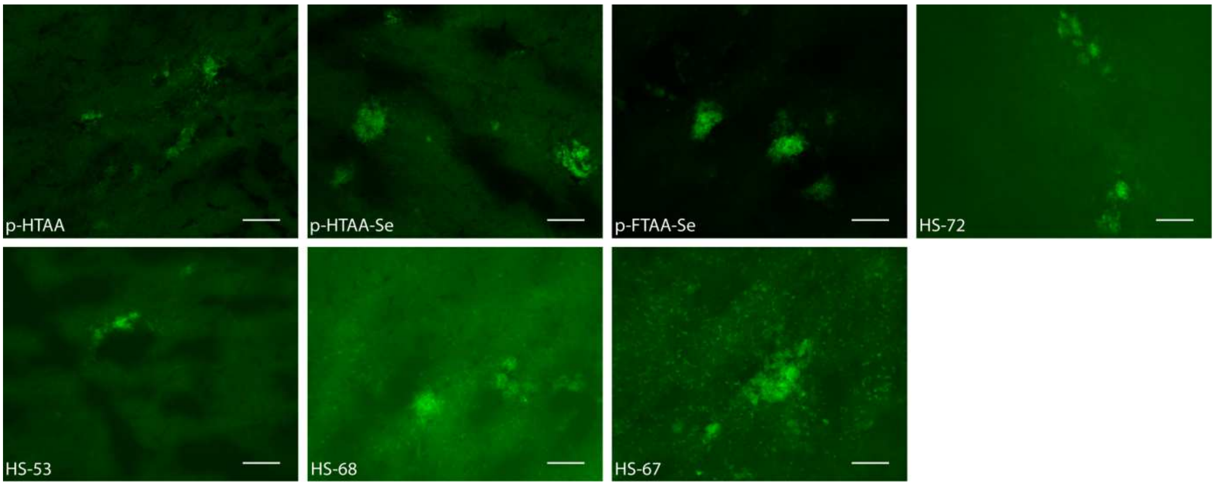

Figure S3: Fluorescence images of p-HTAA, p-HTAA-Se, p-FTAA-Se, HS-72, HS-53, HS-68 and HS-67 stained brain sections from prion infected transgenic mice. Scale bars represent 50 μm.

Table S1. Statistical analysis of spectral difference between Aβ plaques and neurofibrillary tangles

	Unpaired t-test (R^2)	Unpaired t-test (Difference of the mean)	Unpaired t-test with Welch´s correction (R^2)
p-FTAA	0.8901	-0,3959 ± 0,01326	0.9220
p-FTAA-Se	0.3421	-0,09355 ± 0,01237	0.3570
p-HTAA	0.1332	0,1266 ± 0,03079	0.1655
p-HTAA-Se	0.1069	0,06771 ± 0,01866	0.1245
HS-72	0.7529	-0,1535 ± 0,008382	0.7591
HS-53	0.8846	-0,07902 ± 0,002721	0.9026
HS-68	0.4911	-0,5883 ± 0,05710	0.5937
HS-67	0.4372	-0,5052 ± 0,05465	0.5097

EXPERIMENTAL DETAILS

General information. NMR spectra were recorded on a Varian instrument 300 instrument (Varian Inc., Santa Clara, CA, USA) operating at 300 MHz for ^{1}H and 75.4 MHz for ^{13}C, using the residual solvent signal as reference. High-resolution mass spectrometry was performed with an Agilent 6540 Accurate-Mass Quadrupole Time-of-Flight (Q-TOF). GC-MS analysis was performed by HP 6890 gas chromatograph with a HP 5973 mass selective detector. The mass detector was run in the electron impact (EI) mode and the ions were scanned in the total ion current - (TIC). IR spectra were acquired on a Thermo Nicolet Avatar 330 FT-IR instrument and Perkin-Elmer Spectrum 1000 using KBr pellets. Melting points were determined in open capillary tubes on a Stuart® melting point apparatus. Chemicals and solvents were obtained from commercial sources and used as received. THF was distilled from sodium and benzophenone, and 1,4-dioxan, was dried over activated 4 Å molecular sieves. Column chromatography was carried out on silica gel Merck 60 (40–63 µm) and reverse phase chromatography (RP) on Merck LiChroprep® (RP-18).

General procedure for bromination. A solution of N-Bromosuccinimide (1 or 2 equiv.) in DMF (3 mL/mmol) was added drop wise to desired thiophene derivative in DMF (2 mL/1 mmol) at 0 °C. The mixture was allowed to reach r.t. overnight (16h). Subsequently water was added and the product was extracted with DCM (3×30 mL/mmol). The organic phase was washed with water (3×30 mL/mmol), brine (30 mL), dried over $MgSO_4$ and the solvent was evaporated under reduce pressure.

General procedure for Suzuki coupling. PEPPS-IPr (5 mol %) was added to a mixture of the bromo thiophene/selenophene derivatives (1-2 equiv), K_2CO_3 (3 equiv./bromine), the desired boronic acid or pinacol esters (1-2 equiv./bromine) in 1,4-dioxane/methanol (8 : 2, 8 mL/mmol, degassed). The mixture was heated to 70 °C for 20 min then cooled to rt and pH adjusted to 4 by 1M HCl. The residue was extracted with DCM (3×30 mL/mmol), washed with water (3×30 mL/mmol), brine (30 mL) and the combined organic phase was dried over $MgSO_4$. The crude product was either subjected to column chromatography or treated with appropriate solvent to give desired products.

General procedure for methylesters hydrolysis. NaOH (1 M, 1.5 equiv./ester) was added to a solution of the oligothio/selenophene in 1,4-dioxane (7 mL/1 mmol) and heated to 50 °C for 5 h. When precipitation appeared, H_2O was added and the solution was lyophilized.

Methyl 2-(2-(4,4,5,5-tetramethyl-1,3,2-dioxaborolan-2-yl)thiophen-3-yl)acetate (1): This compound was prepared according to described procedure. Pinacolborane (0.817 g/ 6.4 mmol) were added to the mixture of methyl 2-(2-bromothiophen-3-yl)acetate x (1 g/ 4.25 mmol) Pd(P(*t*-Bu)$_3$)$_2$ (0.5 mol %) triethylamine (1.3g/ 12.8 mmol) in dry THF (5 mL/mmol). The mixture was stirred at 40°C under N_2 for 2 h and solvent was removed under reduced pressure. The residue was re-dissolved in THF/H_2O 1:1 (10 mL/mmol) pH adjusted to 4 by acetic acid and extracted with ether (3×30 mL/mmol), washed with water (3×30 mL/mmol), brine (30 mL). The combined organic phase was dried over $MgSO_4$ and the solvent evaporated under reduce pressure giving light-brown oil. The product yellow oil was subjected to column chromatography using oven dried silica [heptane/EtOAc/TEA (10:1→1:1)], yielding the compound 1 (540 mg, 45 %) as colorless oil. (This procedure gives a variation of yields from 45 to 55%)

IR (neat) 1740, 1536, 1032, 962, 856, 783, 730 cm^{-1}. ^{1}H NMR (300 MHz, $CDCl_3$) δ 7.54 (d, *J* = 4.7 Hz, 1H), 7.11 (d, *J* = 4.7 Hz, 1H), 3.99 (s, 2H), 3.69 (s, 3H), 1.32 (s, 12H). ^{13}C NMR (75 MHz, $CDCl_3$) δ 175.6, 144.2, 144.1 131.9, 130.7, 84.0, 52.0, 35.5, 24.9. HRMS (ESI–TOF): *m/z* calcd for $C_{13}H_{19}BO_4S$ $(M+H)^+$ 282,1097 found 282,1173.

Dimethyl 2,2'-(2,2'-(selenophene-2,5-diyl)bis(thiophene-3,2-diyl))diacetate (3): This compound was prepared according to the general procedure of Suzuki coupling starting with 2,5-dibromoselenophene (**2**) (0.15g, 0.52 mmol) and monomer (**1**) (0,293 g, 1 mmol). The crude product was purified by gradient column chromatography using [heptane/EtOAc (8:1→5:1)] to give the product (0.112 g, 49%) as yellow oil.

IR (neat) 1730, 1433, 1243, 1194, 1168, 1011, 806, 706 cm^{-1}. ^{1}H NMR (300 MHz, $CDCl_3$) δ 7.29 (s, 2H), 7.24 (d, *J* = 5.3 Hz, 2H), 7.05 (d, *J* = 5.3 Hz, 2H), 3.78 (s, 2H), 3.73 (s, 3H). ^{13}C NMR (75 MHz, $CDCl_3$) δ 171.4, 141.4, 135.6, 130.5, 130.2, 129.8, 124.9, 52.3, 34.9. HRMS (ESI–TOF): *m/z* calcd for $C_{18}H_{16}O_4S_2Se$ $(M+H)^+$ 439.9655 found 439.9726.

Dimethyl 2,2'-(2,2'-(selenophene-2,5-diyl)bis(5-bromothiophene-3,2-diyl))diacetate (4): General procedure of bromination was applied starting with trimer **3** (0.113 g, 0.26 mmol) and NBS (0.092 g, 0.51 mmol). The crude product yellow oil was purified by column chromatography [heptane/EtOAc (7:1)] to give compound **4** (0.12 g, 78%) as yellow solid; mp 90.5-91.5 °C.

IR (neat) 1727, 1428, 1331, 1247, 1221, 1194, 1122, 989, 894, 801, 760 cm^{-1}. ^{1}H NMR (300 MHz, $CDCl_3$) δ 7.24 (s, 2H), 7.03 (s, 2H), 3.74 (s, 3H), 3.70 (s, 2H). ^{13}C NMR (75 MHz, $CDCl_3$) δ 170.9, 140.5, 136.7, 133.1, 130.8, 130.2, 112.0, 52.5, 34.6. HRMS (ESI–TOF): *m/z* calcd for $C_{18}H_{14}Br_2O_4S_2Se$ $(M+H)^+$ 595.7865 found 595.7929.

Compound 6: General procedure of Suzuki coupling was applied starting with trimer 4 (0.080 g, 0.134 mmol) and compound **5**[1] (0,072 g, 0.27 mmol). The crude product red solid was treated with warm MeOH to give pentamer **6** (0.08 g, 83 %) as red solid; mp 183,5-185 °C.

IR (neat) 1731, 1710, 1698, 1457, 1434, 1284, 1253, 1190, 1096, 786, 744 cm^{-1}. ^{1}H NMR (300 MHz, $CDCl_3$) δ 7.70 (d, *J* = 4.0 Hz, 1H), 7.36 (s, 1H), 7.24 (s, 1H), 7.15 (d, *J* = 4.0 Hz, 1H), 3.90 (s, 3H), 3.77 (s, 2H), 3.77 (s, 3H). ^{13}C NMR (75 MHz, $CDCl_3$) δ 171.0, 162.5, 143.4, 141.0, 135.9, 135.3, 134.4, 132.0, 131.2, 130.2, 128.5, 124.3, 52.6, 52.4, 35.0. HRMS (ESI–TOF): *m/z* calcd for $C_{30}H_{24}O_8S_4Se$ $(M+H)^+$ 719.9519 found 719.9600.

Compound (9): General procedure of Suzuki coupling was applied starting with trimer **7** (0.140 g, 0.25 mmol) and compound **8** (0,131 g, 0.50 mmol). The crude product yellow oil was purified by gradient column chromatography using [heptane/EtOAc (4:1→1:1)] to give pentamer **9** (0.120 g, 73 %) as yellow solid; mp 104-105.5 °C.

IR (neat) 1725, 1431, 1248, 1007, 825, 776, 687 cm^{-1}. ^{1}H NMR (300 MHz, $CDCl_3$) δ 7.90 (dd, *J* = 5.6, 1.1 Hz, 1H), 7.33 (dd, *J* = 3.8, 1.1 Hz, 1H), 7.27 – 7.22 (m, 1H), 7.18 (s, 1H), 7.08 (s, 1H), 3.77 (s, 2H), 3.75 (s, 3H). ^{13}C NMR (75 MHz, $CDCl_3$) δ 171.2, 141.8, 138.7, 135.6, 131.9, 131.1, 130.5, 130.3, 127.6, 127.5, 126.4, 52.5, 35.0. HRMS (ESI–TOF): *m/z* calcd for $C_{26}H_{20}O_4S_3Se_2$ $(M+H)^+$ 651.8854 found 651.8928.

Methyl 2-(2,5-dibromothiophen-3-yl)acetate (13): To a solution of 2-(thiophen-3-yl)acetic acid (5 g, 35.2 mmol) in methanol (40 mL) was add conc. sulfuric acid (35.2 mmol) and the mixture was heated at 70°C for 16 h. After cooling to r.t. the solvent was evaporated under reduced pressure and EtOAc (60 mL) was added. The organic layer was washed 3 times with 5% aq. Na_2CO_3 and the combined aqueous layer washed with more EtOAc (40 mL). The combined organic phases washed with water (3×50 mL), brine (50 mL) and dry with $MgSO_4$. The solvent was removed under reduced pressure and the residue was dried in oil pump vacuum to obtain ester as off-white oil. This was redissolved in DMF (30 mL) and NBS (12.5 g, 70.4 mmol) in DMF (10 mL) was added. The reaction mixture was stirred for 16 h, thereafter water was added and extracted with DCM (3×50 mL), washed with water (3×50 mL), brine (50 mL) and the combined organic phase was dried over $MgSO_4$. The crude product yellow oil was subjected to column chromatography [heptane/EtOAc (8:1)], yielding the product **13** (9.7 g, 88 %) as colorless oil which solidifies upon standing.

IR (neat) 1729, 1436, 1429, 1334, 1193, 1168, 1020, 994, 975, 891, 830, 736 cm^{-1}. ^{1}H NMR (300 MHz, CD_3OD) δ 7.01 (s, 1H), 3.69 (s, 3H), 3.62 (s, 2H). ^{13}C NMR (75 MHz, CD_3OD) δ 171.7, 136.6, 133.2, 111.7, 111.6, 52.7, 35.3.

Methyl 2-([2,2'-bithiophen]-3-yl)acetate (14): This compound was prepared according to the general procedure of Suzuki coupling starting with compound **10** (0.260 g, 1.24 mmol) and **12** (0.292 g, 1.24 mmol). Gradient column chromatography [heptane/EtOAc (10:1→6:1)] gave dimer **14** as yellow oil (0.230g, 78%).

IR (neat) 1732, 1434, 1265, 1244, 1194, 1166, 1012, 847, 829, 696, 638 cm^{-1}. H NMR (300 MHz, DMSO) δ 7.60 (dd, *J* = 5.1, 1.3 Hz, 1H), 7.49 (d, *J* = 5.2 Hz, 1H), 7.20 (dd, *J* = 3.6, 1.2 Hz, 1H), 7.14 (dd, *J* = 5.1, 3.6 Hz, 1H), 7.07 (d, *J* = 5.2 Hz, 1H), 3.79 (s, 2H), 3.63 (s, 3H). ^{13}C NMR (75 MHz, DMSO) δ 170.8, 134.4, 132.3, 130.9, 130.5, 128.1, 126.8, 126.4, 124.8, 51.8, 33.9. HRMS (ESI–TOF): *m/z* calcd for $C_{11}H_{10}O_2S_2$ $(M+H)^+$ 238,0122 found 238,0195.

Methyl 2-(2-(selenophen-2-yl)thiophen-3-yl)acetate (15): This compound was prepared according to the general procedure of Suzuki coupling starting with compound **11** (0.257g, 1 mmol) and **12** (0,235 g, 1 mmol). The crude product, yellow oil, was purified by gradient column chromatography [heptane/EtOAc (10:1→6:1)] to give dimer **15** (0.18 g, 73%) as yellow oil.

IR (neat) 1731, 1433, 1263, 1241, 1194, 1169, 1011, 832, 780, 684, 649 cm^{-1}. ^{1}H NMR (300 MHz, DMSO) δ 8.23 (dd, *J* = 5.5, 1.3 Hz, 1H), 7.48 (d, *J* = 5.2 Hz, 1H), 7.38 – 7.29 (m, 2H), 7.06 (d, *J* = 5.2 Hz, 1H), 3.77 (s, 2H), 3.63 (s, 3H). ^{13}C NMR (75 MHz, DMSO) δ 170.8, 139.5, 134.8, 133.2, 131.0, 130.2, 130.2, 130.0, 128.6, 124.8, 51.8, 34.0. HRMS (ESI–TOF): *m/z* calcd for $C_{11}H_{10}O_2SSe$ $(M+H)^+$ 285,9567 found 285,9641.

Methyl 2-(5,5'-dibromo-[2,2'-bithiophen]-3-yl)acetate (16): General procedure of bromination was applied starting with compound **14** (0.366 g, 1.54 mmol) and NBS (0.547 g, 3.1 mmol). The crude product was purified by column chromatography [heptane/EtOAc (7:1)] to give dimer **16** as yellow solid (0.52 g, 85%); mp: 49-50 °C.

IR (neat) 1730, 1430, 1414, 1337, 1315, 1214, 1198, 1143, 987, 892, 792, 757 cm^{-1}. ^{1}H NMR (300 MHz, DMSO) δ 7.28 (d, *J* = 3.9 Hz, 1H), 7.22 (s, 1H), 7.05 (d, *J* = 3.9 Hz, 1H), 3.75 (s, 2H), 3.64 (s, 3H). ^{13}C NMR (75 MHz, DMSO) δ 170.3, 134.5, 134.0, 132.7, 132.4, 131.4, 128.2, 112.5, 110.5, 52.0, 33.6. HRMS (ESI–TOF): *m/z* calcd for $C_{11}H_8Br_2O_2S_2$ $(M+H)^+$ 393.8332 found 393.8408.

Methyl 2-(5-bromo-2-(5-bromoselenophen-2-yl)thiophen-3-yl)acetate (17): General procedure of bromination was applied starting with compound **15** (0.332 g, 1.16 mmol) and NBS (0.414 g, 2.33 mmol). The crude product yellow oil was purified by column chromatography [heptane/EtOAc (7:1)] to give dimer **17** (0.45 g, 88%) as yellow solid; mp: 37-38 °C.

IR (neat) 1733, 1422, 1259, 1195, 1169, 1124, 931, 833, 794 cm^{-1}. ^{1}H NMR (300 MHz, DMSO) δ 7.45 (d, *J* = 4.1 Hz, 1H), 7.21 (s, 1H), 7.12 (d, *J* = 4.1 Hz, 1H), 3.73 (s, 2H), 3.64 (s, 3H). ^{13}C NMR (75 MHz, DMSO) δ 170.3, 139.8, 135.4, 134.1, 134.0, 131.9, 130.1, 116.7, 110.4, 52.0, 40.3, 40.1, 39.8, 39.5, 39.2, 39.0, 38.7, 33.6. HRMS (ESI–TOF): *m/z* calcd for $C_{11}H_8Br_2O_2SSe$ $(M+H)^+$ 441.7777 found 441.7842.

Compound (18): General procedure of Suzuki coupling was applied starting with dimer **16** (0.420g, 1.10 mmol) and compound **5** (0,312 g, 1.20 mmol). The product was recrystallized from ACN to give tetramer **18** (0.260 g, 47%) as red solid; mp 163.5-165 °C.

IR (neat) 1732, 1693, 1445, 1299, 1276, 1188, 1093, 785, 745 cm^{-1}. ^{1}H NMR (300 MHz, $CDCl_3$) δ 7.71 – 7.68 (m, 2H), 7.26 – 7.13 (m, 5H), 3.90 (s, 3H), 3.89 (s, 3H), 3.78 (s, 2H), 3.76 (s, 3H). ^{13}C NMR (75 MHz, $CDCl_3$) δ 170.9, 162.5, 143.5, 143.3, 137.4, 135.4, 135.1, 134.5, 134.4, 133.4, 132.0, 132.0, 131.7, 128.5, 128.1, 126.0, 124.4, 124.4, 77.6, 77.2, 76.7, 52.6, 52.4, 34.9. HRMS (ESI–TOF): *m/z* calcd for $C_{23}H_{18}O_6S_4$ $(M+H)^+$ 517.9986 found 518.0064.

Compound (19): General procedure of Suzuki coupling was applied starting with dimer **17** (0.375g, 0.85 mmol) and compound **5**[1] (0,250 g, 0.931 mmol). The product was recrystallized from ACN to give tetramer **19** (0.21 g, 44%) as red solid; mp 160-161 °C.

IR (neat) 1729, 1697, 1441, 1296, 1284, 1176, 1099, 928, 785, 745 cm^{-1}. ^{1}H NMR (300 MHz, $CDCl_3$) δ 7.70 (dd, *J* = 3.9, 1.7 Hz, 2H), 7.42 (d, *J* = 4.0 Hz, 1H), 7.31 (d, *J* = 4.0 Hz, 1H), 7.23 (s, 1H), 7.15 (d, *J* = 3.9 Hz, 1H), 7.11 (d, *J* = 3.9 Hz, 1H), 3.90 (s, 6H), 3.76 (s, 5H). ^{13}C NMR (75 MHz, $CDCl_3$) δ 170.9, 162.6, 162.5, 145.8, 143.3, 142.9, 139.9, 135.8, 135.4, 134.5, 134.4, 132.0, 132.0, 131.3, 130.4, 128.5, 128.1, 124.9, 124.4, 52.6, 52.4, 34.9. HRMS (ESI–TOF): *m/z* calcd for $C_{23}H_{18}O_6S_3Se$ $(M+H)^+$ 565.9431 found 565.9510.

Methyl 2-([2,2':5',2''-terthiophen]-3'-yl)acetate (20): This compound was prepared according to the general procedure of Suzuki coupling starting with compound **13** (0.90 g, 3 mmol) and compound **10** (1.26 g, 6 mmol). The crude product was purified by gradient column chromatography [heptane/EtOAc (10:1→5:1)] to give trimer **20** (0.83 g, 86%) as yellow solid; mp 46-47 °C.

IR (neat) 1739, 1436, 1411, 1315, 1195, 1166, 829, 705 cm^{-1}. ^{1}H NMR (300 MHz, DMSO) δ 7.64 (dd, *J* = 5.1, 1.2 Hz, 1H), 7.54 (dd, *J* = 5.1, 1.1 Hz, 1H), 7.32 (dd, *J* = 3.6, 1.2 Hz, 1H), 7.24 (m, 3H), 7.16 (dd, *J* = 5.1, 3.6 Hz, 2H), 7.10 (dd, *J* = 5.1, 3.6 Hz, 1H), 3.81 (s, 1H), 3.65 (s, 1H). ^{13}C NMR (75 MHz, DMSO) δ 170.6, 135.6, 134.4, 133.9, 131.3, 131.1, 128.4, 128.2, 127.5, 127.1, 126.6, 125.8, 124.3, 51.9, 34.0. HRMS (ESI–TOF): *m/z* calcd for $C_{15}H_{12}O_2S_3$ $(M+H)^+$ 319.9999 found 320.0078.

Methyl 2-(2,5-di(selenophen-2-yl)thiophen-3-yl)acetate (21): This compound was synthesized following same procedure as for compound **20** on a 0.65 mmol scale with compound **11** instead of **10**. Same column chromatography system gave trimer **21** as yellow solid (0.22g, 81%); mp 54.5-56 °C.

IR (neat) 1733, 1434, 1309, 1192, 1163, 836, 771, 737, 686 cm^{-1}. ^{1}H NMR (300 MHz, $CDCl_3$) δ 8.04 (dd, *J* = 5.4, 1.3 Hz, 1H), 7.89 (dd, *J* = 5.6, 1.1 Hz, 1H), 7.38 -7.20 (m, 4H), 7.07 (s, 1H), 3.74 (s, 3H), 3.72 (s, 2H). ^{13}C NMR (75 MHz, $CDCl_3$) δ 170.3, 140.9, 138.8, 137.4, 133.9, 131.3, 129.5, 129.5, 129.4, 129.1, 128.3, 126.5, 125.2, 51.4, 33.9. HRMS (ESI–TOF): *m/z* calcd for $C_{15}H_{12}O_2SSe_2$ $(M+H)^+$ 415.8888 found 415.8962.

Methyl 2-(5,5''-dibromo-[2,2':5',2''-terthiophen]-3'-yl)acetate (22): General procedure of bromination was applied starting with compound **20** (0.50 g, 1.56 mmol) and NBS (0.560 g, 3.1 mmol). The crude product yellow oil was purified by column chromatography [heptane/EtOAc (8:1)] to give trimer **22** (0.67 g, 90 %) as yellow solid; mp 86-87.5 °C.

IR (neat) 1729, 1506, 1430, 1422, 1334, 1203, 1175, 1150, 790 cm^{-1}. ^{1}H NMR (300 MHz, DMSO) δ 7.30 (d, *J* = 3.9 Hz, 1H), 7.26 (s, 1H), 7.24 (d, *J* = 3.9 Hz, 1H), 7.19 (d, *J* = 3.9 Hz, 1H), 7.09 (d, *J* = 3.9 Hz, 1H), 3.78 (s, 2H), 3.65 (s, 3H). ^{13}C NMR (75 MHz, DMSO) δ 170.4, 137.0, 135.3, 133.8, 132.3, 131.7, 131.5, 130.4, 128.1, 127.7, 125.2, 112.1, 110.9, 52.0, 33.9. HRMS (ESI–TOF): *m/z* calcd for $C_{15}H_{10}Br_2O_2S_3$ $(M+H)^+$ 475.8210 found 475.8276.

Methyl 2-(2,5-bis(5-bromoselenophen-2-yl)thiophen-3-yl)acetate (23): General procedure of bromination was applied starting with compound **21** (0.180 g, 0.44 mmol) and NBS (0.157 g, 0.88 mmol). The crude product yellow oil was purified by column chromatography [heptane/EtOAc (8:1)] to give trimer **23** (0.22 g, 88%) as yellow solid; mp 91-92 °C.

IR (neat) 1744, 1509, 1428, 1342, 1149, 958, 932, 819, 781 cm^{-1}. ^{1}H NMR (300 MHz, $CDCl_3$) δ 7.24 (d, *J* = 4.1 Hz, 1H), 7.17 (d, *J* = 4.1 Hz, 1H), 7.04 (d, *J* = 4.1 Hz, 1H), 7.00 (d, *J* = 4.1 Hz, 1H), 6.97 (s, 1H), 3.74 (s, 4H), 3.67 (s, 2H). ^{13}C NMR (75 MHz, $CDCl_3$) δ 171.0, 143.4, 141.3, 137.9, 134.3, 133.9, 133.7, 131.1, 129.5, 127.5, 126.2, 117.3, 115.1, 52.5, 34.8. HRMS (ESI–TOF): *m/z* calcd for $C_{15}H_{10}Br_2O_2SSe_2$ $(M+H)^+$ 571.7099 found 571.7158.

Compound 24: General procedure of Suzuki coupling was applied starting with trimer **22** (0.420 g, 0.878 mmol) and compound **5** (0,483 g, 1.80 mmol). The product was treated with warm EtOAc to give pentamer **24** (0.430 g, 82 %) as red solid; mp 186-187.5 °C.

IR (neat) 1729, 1695, 1442, 1287, 1254, 1189, 1095, 1064, 844, 795, 779, 742 cm^{-1}. ^1H NMR (300 MHz, $CDCl_3$) ^1H NMR (300 MHz, $CDCl_3$) δ 7.70 (dd, *J* = 4.0, 1.8 Hz, 2H), 7.25 – 7.09 (m, 7H), 3.90 (s, 6H), 3.78 (s, 2H), 3.76 (s, 3H). ^{13}C NMR (75 MHz, $CDCl_3$) δ 171.0, 162.6, 143.8, 143.6, 137.2, 137.1, 135.8, 135.7, 135.4, 134.5, 132.3, 131.9, 131.7, 131.6, 127.9, 127.5, 126.1, 126.0, 125.1, 124.3, 124.1, 52.5, 52.4, 35.0. HRMS (ESI–TOF): *m/z* calcd for $C_{27}H_{20}O_6S_5$ $(M+H)^+$ 599.9863 found 599.9950.

Compound 25: General procedure of Suzuki coupling was applied starting with trimer **23** (0.100 g, 0.18 mmol) and compound **5** (0,094 g, 0.35 mmol). The crude product red oil was purified by gradient column chromatography using [heptane/EtOAc (4:1→2:1)] and then treated with warm MeOH to give pentamer **25** (0.073 g, 60 %) as red solid; mp 176-177 °C.

IR (neat) 1563, 1430, 1375, 803, 782, 770 cm^{-1}. ^1H NMR (300 MHz, $CDCl_3$) δ 7.69 (dd, *J* = 3.9, 1.8 Hz, 2H), 7.42 (d, *J* = 4.0 Hz, 1H), 7.35 (d, *J* = 4.0 Hz, 1H), 7.30 (d, *J* = 4.0 Hz, 1H), 7.26 (d, *J* = 4.0 Hz, 1H), 7.11 – 7.06 (m, 3H), 3.90 (s, 3H), 3.90 (s, 3H), 3.76 (s, 3H), 3.75 (s, 2H). ^{13}C NMR (75 MHz, $CDCl_3$) δ 171.0, 162.6, 146.0, 145.9, 142.6, 142.2, 140.5, 140.2, 138.1, 134.9, 134.5, 132.0, 131.8, 131.2, 130.1, 128.3, 128.2, 128.1, 127.1, 124.9, 124.7, 52.6, 52.4, 35.0. HRMS (ESI–TOF): *m/z* calcd for $C_{27}H_{20}O_6S_3Se_2$ $(M+H)^+$ 695.8752 found 695.8831.

Dimethyl 2,2'-(2,2'-(1,4-phenylene)bis(thiophene-3,2-diyl))diacetate (26): 12 (0.20 g, 0.85 mmol) and *p*-phenylenediboronic acid (0.20 g, 1,7 mmol) was subjected to the conditions of general procedure for Suzuki coupling. The product was recrystallized from DMSO to give product **26** (0.25 g, 76%) as white crystals; mp 130.5-134.5 °C.

IR (neat) 3109, 1745, 1434, 1273, 1230, 1016, 845, 725. ^1H NMR (300 MHz, $CDCl_3$) δ 7.53 (s, 4H), 7.30 (d, *J* = 5.2 Hz, 2H), 7.10 (d, *J* = 5.2 Hz, 2H), 3.73 (s, 6H), 3.71 (s, 4H). ^{13}C NMR (75 MHz, $CDCl_3$) δ 171.9, 140.3, 133,4, 130.1, 129.8, 129.7, 124.6, 52.3, 34.5. HRMS (ESI–TOF): *m/z* calcd for $C_{20}H_{18}O_4S_2$ $(M+H)^+$ 386.0647 found 386.0720

Dimethyl 2,2'-(2,2'-(1,4-phenylene)bis(5-bromothiophene-3,2-diyl))diacetate (27): The general procedure for brominations was applied to **26** (0.21 g, 0.54 mmol) and NBS (0.19, 1.1 mmol). After reaction the product was purified by flash chromatography to give **27** (0.27 g, 91 %) as a white powder; mp 160.7-162.0 °C.

IR (neat) 1749, 1557, 1500, 1434, 1277, 1272, 1246, 1231, 1170, 1015, 845, 725 cm^{-1}. ^1H NMR (300 MHz, $CDCl_3$) δ 7.47 (s, 4H), 7.06 (s, 2H), 3.74 (s, 6H), 3.62 (s, 4H),. ^{13}C NMR (75 MHz, $CDCl_3$) δ 171.3, 141.6, 132.8, 132.7, 130.3, 129.8, 111.6, 52.4, 34.3. HRMS (ESI–TOF): *m/z* calcd for $C_{20}H_{16}Br_2O_4S_2$ $(M+H)^+$ 541.8857 found 541.8920

Dimethyl 2,2'-(2,2'-(2,5-dimethoxy-1,4-phenylene)bis(thiophene-3,2-diyl))diacetate (29):1,4-Bis-(4,4,5,5-tetramethyl[1,3,2]dioxaborolan)-2,5 dimethoxybenzene (**28**) was synthesized according to procedures previously described by Glasson et al.[2] and NMR was agreeing to those reported. **12** (0.72 g, 3.1 mmol) and **28** (0.40 g, 1,0 mmol) was subjected to the conditions of general procedure for Suzuki coupling. The product was purified on flash chromatography (toluene/EtOAc) to give product **29** (0.11 g, 24%) as white crystals; mp 132.5-134.0 °C.

IR (neat) 1721, 1504, 1466, 1415, 1377, 1217, 1196, 1156, 1049, 861, 778, 707 cm^{-1}. ^1H NMR (300 MHz, $CDCl_3$) δ 7.35 (d, *J* = 5.2 Hz, 1H), 7.12 (d, *J* = 5.2 Hz, 1H), 6.99 (s, 1H), 3.74 (s, 3H), 3.70 (s, 3H), 3.58 (s, 2H). ^{13}C NMR (75 MHz, $CDCl_3$) δ 171.9, 150.6, 135.6, 131.4, 129.3, 124.9, 122.9, 115.7, 56.1, 51.9, 34.7. HRMS (ESI–TOF): *m/z* calcd for $C_{22}H_{22}O_6S_2$ $(M+H)^+$ 446.0858 found 446.0930

Dimethyl 2,2'-(2,2'-(2,5-dimethoxy-1,4-phenylene)bis(5-bromothiophene-3,2-diyl))diacetate (30): The general procedure for brominations was applied to **29** (0.11 g, 0.24 mmol) and NBS (0.85, 0.48 mmol). The product was purified by recrystallizing the crude product from acetonitrile to give **30** as a white powder (0.83 mg, 56%); mp 200-201 °C.

IR (neat) 1735, 1502, 1464, 1432, 1380, 1265, 1233, 1147, 1044, 1006, 869, 783 cm^{-1}. ^1H NMR (300 MHz, $CDCl_3$) δ 7.08 (s, 1H), 6.95 (s, 1H), 3.75 (s, 3H), 3.71 (s, 3H), 3.50 (s, 2H). ^{13}C NMR (75 MHz, $CDCl_3$) δ 171.5, 150.7, 137.1, 132.1, 132.1, 122.4, 115.5, 112.0, 56.3, 52.2, 34.8. HRMS (ESI–TOF): *m/z* calcd for $C_{22}H_{20}Br_2O_6S_2$ $(M+H)^+$ 601.9068 found 601.9139

p-FTAA-Se: Pentamer **6** (0.05 g, 0.07 mmol) was hydrolyzed according to the general procedure to give the final product as red solid (calculated yield for free acid: 0.046 g, 100%); mp >300 °C.

IR (neat) 1560, 1458, 1386 cm^{-1}. ^1H NMR (300 MHz, D_2O) δ 7.41 (dd, *J* = 3.9, 0.7 Hz, 1H), 7.26 (d, *J* = 0.5 Hz, 1H), 7.11 (s, 1H), 7.10 (dd, *J* = 3.9, 0.5 Hz, 1H), 3.67 (s, 2H). ^{13}C NMR (75 MHz, D_2O) δ 179.2, 169.8, 140.9, 140.4, 139.5, 134.5, 134.3, 134.2, 132.0, 129.2, 128.5, 124.3, 38.5. HRMS (ESI–TOF): *m/z* calcd for $C_{26}H_{16}O_8S_4Se$ $(M-H)^-$ 663.8893 found 663.8813.

p-HTAA-Se: Pentamer **9** (0.06 g, 0.062 mmol) was hydrolyzed according to the general procedure to give the final product as yellow solid (calculated yield for free acid: 0.056 g, 97%); mp >300 °C.

IR (neat) 1592, 1502, 1432, 1393, 1276, 862, 820, 774 cm^{-1}. ^1H NMR (300 MHz, D_2O) 7.89 (d, *J* = 5.6 Hz, 1H), 7.26 – 7.09 (m, 2H), 6.96 (s, 1H), 6.92 (s, 1H), 3.51 (s, 2H). ^{13}C NMR (75 MHz, D_2O) δ 179.2, 141.6, 136.9, 135.1, 134.1, 130.9, 130.7, 130.1, 128.4, 126.2, 125.9, 38.2. HRMS (ESI–TOF): *m/z* calcd for $C_{24}H_{16}O_4S_3Se_2$ $(M+H)^+$ 623.8541 found 623.8611.

HS-67: Tetramer **19** (0.21 g, 0.371 mmol) was hydrolyzed according to the general procedure to give HS-67 as red solid (calculated yield for free acid: 0.19 g, 100%); mp >300 °C.

IR (neat) 1568, 1445, 1376, 803, 770 cm^{-1}. ^1H NMR (300 MHz, D_2O) δ 7.57 (d, *J* = 4.1 Hz, 1H), 7.53 (t, *J* = 4.1 Hz, 2H), 7.40 (d, *J* = 4.1 Hz, 1H), 7.32 (m, 3H), 3.74 (s, 3H). ^{13}C NMR (75 MHz, D_2O) δ 178.9, 169.6, 142.9, 141.8, 140.6, 139.6, 139.6, 139.5, 134.6, 134.2, 134.1, 131.8, 131.7, 129.1, 128.8, 127.4, 124.9, 124.2. HRMS (ESI–TOF): *m/z* calcd for $C_{20}H_{12}O_6S_3Se$ $(M-H)^-$ 523.8961 found 523.8887.

HS-68: Tetramer **18** (0.1 g, 0.193 mmol) was hydrolyzed according to the general procedure to give HS-68 as red solid (calculated yield for free acid: 0.09 g, 98%); mp >300 °C.

IR (neat) 1568, 1449, 1376, 803, 770 cm^{-1}. ^1H NMR (300 MHz, D_2O) δ 7.48 (d, *J* = 3.9 Hz, 2H), 7.33 (d, *J* = 3.9 Hz, 1H), 7.25 – 7.21 (m, 3H), 7.17 (d, *J* = 3.9 Hz, 1H), 3.72 (s, 2H). ^{13}C NMR (75 MHz, D_2O) δ 179.1, 169.6, 140.6, 140.6, 139.4, 136.3, 135.1, 134.7, 134.2, 131.7, 131.7, 129.0, 126.6, 125.3, 124.2, 124.2, 38.1. HRMS (ESI–TOF): *m/z* calcd for $C_{20}H_{12}O_6S_4$ $(M-H)^-$ 475.9517 found 475.9445.

HS-53: Pentamer **25** (0.050 g, 0.072 mmol) was hydrolyzed according to the general procedure to give HS-53 as red solid (calculated yield for free acid: 0.046 g, 97%); mp >300 °C.

IR (neat) 1568, 1430, 1381, 804, 771 cm^{-1}. ^1H NMR (300 MHz, D_2O) δ 7.38 (td, *J* = 3.8, 0.8 Hz, 2H), 7.30 (d, *J* = 3.8 Hz, 1H), 7.24 (d, *J* = 4.1 Hz, 1H), 7.14 (d, *J* = 4.1 Hz, 1H), 7.04 – 6.98 (m, 3H), 3.64 (s, 2H). ^{13}C NMR (75 MHz, D_2O) δ 178.8, 169.5, 169.4, 143.0, 142.9, 141.3, 141.0, 140.6, 140.1, 139.7, 139.4, 136.1, 134.6, 134.3, 133.9, 131.8, 131.7, 129.3, 128.4, 127.8, 127.4, 126.8, 124.6, 38.4. HRMS (ESI–TOF): *m/z* calcd for $C_{24}H_{14}O_6S_3Se_2$ $(M-H)^-$ 653.8283 found 653.8214.

HS-72: Pentamer **24** (0.150 g, 0.250 mmol) was hydrolyzed according to the general procedure to give HS-72 as red solid (calculated yield for free acid: 0.139 g, 100%); mp >300 °C.

IR (neat) 1568, 1430, 1381, 804, 771 cm^{-1}. ^1H NMR (300 MHz, D_2O) δ 7.36 (dd, *J* = 6.6, 3.8 Hz, 2H), 7.02 (d, *J* = 3.8 Hz, 1H), 6.99 (s, 1H), 6.98 – 6.84 (m, 5H), 3.63 (s, 1H). ^{13}C NMR (75 MHz, d_2o) δ 178.9, 169.5, 169.4, 140.7, 140.7, 139.1, 139.1, 136.0, 135.7, 135.2, 135.1, 134.1, 133.8, 131.7, 131.7, 131.2, 128.2, 126.1, 125.4, 125.0, 124.6, 123.9, 123.8, 38.7. HRMS (ESI–TOF): *m/z* calcd for $C_{24}H_{14}O_6S_5$ $(M-H)^-$ 557.9394 found 557.9323.

p-FTAA-Ph: **27** (0.10 g, 0.18 mmol) and 5-boronothiophene-2-carboxylic acid (0.063 g, 0.37 mmol) were subjected to the Suzuki coupling procedure. The hydrolysis procedure were applied to the crude product before it was purified on HPLC (Acetonitrile/H_2O, with 0.05% triethylamine) to give **p-FTAA-Ph** (0,090 g, 80%) as a yellow powder; mp > 300 °C.

IR (neat) 2972, 2650, 1700, 1669, 1458, 1435, 1228, 1099, 748. ^1H NMR (300 MHz, D_2O) δ 7.56 (d, *J* = 0.7 Hz, 2H), 7.50 (dd, *J* = 3.8, 0.7 Hz, 1H), 7.29 (d, *J* = 0.7 Hz, 1H), 7.25 (dd, *J* = 3.8, 0.7 Hz, 1H), 3.63 (s, 2H). ^{13}C NMR (75 MHz, D_2O) δ 179.9, 169.7, 141.0, 139.3, 138.38, 134.7, 134.3, 132.7, 131.8, 128.8, 128.6, 124.1, 37.6. HRMS (ESI–TOF): *m/z* calcd for $C_{30}H_{22}O_{10}S_4$ $(M-H)^-$ 670.0096 found 670.0075

p-FTAA-MeOPh: **30** (0.070 g, 0.12 mmol) and 5-boronothiophene-2-carboxylic acid (0.048 g, 0.24 mmol) were subjected to the Suzuki coupling procedure. The hydrolysis procedure was applied to the crude product before it was purified on HPLC (Acetonitrile/H_2O, with 0.05% triethylamine) to give p-FTAA-MeOPh (0,074 g, 82%) as a yellow powder; mp >300 °C

IR (neat) 3385, 1575, 1455, 1209, 1038, 880, 772 cm^{-1}. ^1H NMR (300 MHz, D_2O) δ 7.50 (d, *J* = 3.8 Hz, 1H), 7.31 (s, 1H), 7.23 (d, *J* = 3.8 Hz, 1H), 7.12 (s, 1H), 3.79 (s, 3H), 3.45 (s, 2H). ^{13}C NMR (75 MHz, CD_3OD/D_2O 1:1) δ 180.0, 170.2, 169.7, 151.7, 142.0, 141.4, 137.6, 136.6, 134.7, 132.3, 128.3, 124.7, 124.0, 116.7, 57.2, 40.0. HRMS (ESI–TOF): *m/z* calcd for $C_{26}H_{18}O_8S_4$ $(M-H)^-$ 609.9885 found 609.9819

Fibrillation of recombinant Aβ1-42: The fibrillation protocol of Aβ1-42 has recently been published elsewhere. Briefly, recombinant Aβ1-42 peptide lyophilized in hydroxyfluoroisopropanol (rPeptide, Athens, GA, USA) was dissolved in 2 mM NaOH to a stock concentration of 1 mg/ml. For the kinetic experiment, the solution of Aβ1-42 was diluted with PBS (10 mM phosphate, 140 mM NaCl, 2.7 mM KCl, pH 7.4) to a final concentration of 10 μM and added to the wells of a microtiter plate (Corning) together with 300 nM ThT, LCO or thiophene-selenophene/phenylene/MeO-phenylene co-oligomer. The plate was incubated at 37°C in quiescent mode and the emission spectrum for each probe was collected every 30 minute using excitation wavelength 440 nm for HS-68, HS-67, 450 nm for p-HTAA, p-HTAA-Se, p-FTAA, p-FTAA-Se, HS-72 and HS-53 and 410 nm for p-FTAA-Ph and p-FTAA-MeOPh. The reference probe ThT was excited at either 410, 440 or 450 nm depending on the concomitantly analyzed LCO or co-oligomer. The kinetic graphs illustrate the fluorescence intensity at the emission wavelength where the maximum peak is found when the probe is binding to Aβ1-42 fibrils (HS-68: 512 nm, HS-67: 522 nm, p-HTAA: 491 nm, p-HTAA-Se: 500 nm, p-FTAA: 508 nm, p-FTAA-Se: 519 nm, HS-72: 511 nm, HS-53: 529 nm, p-FTAA-Ph: 495 nm, p-FTAA-MeOPh: 495 nm and ThT: 485 nm) plotted against time. The same emission wavelengths were also used when collecting the excitation spectrum for each probe in PBS buffer or ThT positive Aβ1-42 fibrils. LCOs and thiophene/selenophene co-oligomers were diluted to a final concentration of 300 nM in PBS to obtain the excitation and emission spectrum for the free probe using the same settings as described above.

Brain tissue collection, processing, and neuropathological assessment: Frozen and formalin-fixed, paraffin wax-embedded brain tissues from clinically and neuropathologically well-characterized cases of AD were obtained from the Knight Alzheimer's Disease Research Center, Washington University School of Medicine, St. Louis, Missouri, USA, using established protocols.[3] Tissues from the middle frontal gyrus of 2 cases were used for this study. The pattern and distribution of inclusions in these cases was previously assessed using consensus criteria for the neuropathologic assessment of AD.[4] Tissue was removed according to Washington University Local Ethics Committee guidelines and informed consent for brain donation was obtained from the next-of- kin.

For the experiments using prion-infected brain slices, we used transgenic mice that develop spontaneous prion disease with prion plaques and spongiform encephalopathy due to a single residue exchange at position 167 (D167S) of the prion protein.[5] Brain was collected from mice at terminal disease and frozen in OCT. For the experiment using brain slices with

aggregated tau pathology, we used transgenic expressing the 383 aa isoform of human tau with the P301S mutation.[6] Brain was collected from mice at 5-6 months of age.

LCO or thiophene-selenophene/phenylene/MeO-phenylene co-oligomer staining of AD frozen brain sections: Frozen brain sections from AD patient or transgenic mice harboring PrP^{D167S} or tau^{P301S} mutation were fixed with 96% EtOH for 10 min. The tissue was rehydrated in 50% EtOH (2 min) and distilled water (2 x 2 min) followed with an incubation step in PBS for 10 min. The LCOs and thiophene-selenophene/phenylene/MeO-phenylene co-oligomers were diluted to 3 μM in PBS and added to the sections. After 30 min of incubation, excessive staining solution was removed with repetitive washing in PBS and the sections were mounted with Dako Fluorescence Mounting Medium (Dako, Glostrup, Denmark). The mounting medium was allowed to solidify over night before the rims were sealed with nail polish and the staining result was analyzed using an inverted LSM 780 confocal microscope (Carl Zeiss, Oberkochen, Germany) or a Leica DM6000 B fluorescence microscope (Leica Microsystems, Wetzlar, Germany) equipped with a SpectraCube module (Applied Spectral Imaging, Migdal, Ha-Emek, Israel). Emission spectra for each probe binding to Aβ plaques or NFTs were collected using single excitation wavelength 405 nm or double excitation wavelengths 405 + 538 nm. When the dual excitation mode is used, the contribution of each wavelength is combined in the emission spectrum, which renders it possible to detect even subtle differences in color.

Statistical methods: For each LCO and thiophene/selenophene co-oligomer, the emission spectra for the histological staining experiments represent the mean emission spectrum for 8 regions of interest in 7 Aβ plaques and 7 NFTs, respectively, yielding in total 56 spectra for each protein entity. The merged emission spectra were used to calculate the ratio of emission intensity at 521 and 556 nm ($521/556_R$) for p-FTAA, 512 and 565 nm ($512/565_R$) for p-FTAA-Se, 495 and 565 nm ($495/565_R$) for p-HTAA, 504 and 556 nm ($504/556_R$) for p-HTAA-Se, 512 and 556 nm ($512/556_R$) for HS-72, 512 and 574 nm ($512/574_R$) for HS-53, 486 and 556 nm ($486/556_R$) for HS-68 and 495 and 565 nm ($495/565_R$) for HS-67. The obtained values for each probe binding to Aβ plaques or NFTs were summarized in a plot and the standard deviation for each set was recorded. To investigate if the merged emission spectra for Aβ plaques or NFTs were significantly different the plotted values were analyzed using the unpaired t test with 99% confidence level (GraphPad, La Jolla, CA, USA).

REFERENCES

(1) Kawamorita, S; Ohmiya, H; Sawamura, M. *J. Org. Chem.* **2010**, *75*, 3855.

(2) Glasson, C. R. K.; Meehan, G. V.; Motti, C. A.; Clegg, J. K.; Turner, P.; Jensen, P.; Lindoy, L. F. *Dalton Trans*, **2011**, *40*, 10481.

(3) Cairns, N. J.; Taylor-Reinwald, L.; Morris. J. C.; & the Alzheimer's Disease Neuroimaging Initiative. *Alzheimer's and Dementia* **2010**, 6, 274.

(4) Consensus recommendations for the postmortem diagnosis of Alzheimer's disease. The National Institute on Aging, and Reagan Institute Working Group on Diagnostic Criteria for the Neuropathological Assessment of Alzheimer's Disease. *Neurobiol Aging* **1997**, *18*, S1-S2.

(5) Sigurdson, C. J.; Joshi-Barr, S.; Bett, C.; Winson, O.; Manco, G.; Schwarz, P.; Rülicke, T.; Nilsson, K. P. R.; Margalith, I.; Raeber, A.; Peretz, D.; Hornemann, S.; Wüthrich, K.; Aguzzi, A. *J Neurosci.* **2011**, *31*, 13840.

(6) Allen, B.; Ingram, E.; Takao, M.; Smith, M. J.; Jakes, R.; Virdee, K.; Yoshida, H.; Holzer, M.; Craxton, M.; Emson, P. C.; Atzori, C.; Migheli, A.; Crowther, R. A.; Ghetti, B.; Spillantini, M. G.; Goedert, M. *J. Neurosci.* **2002**, *22*, 9340.

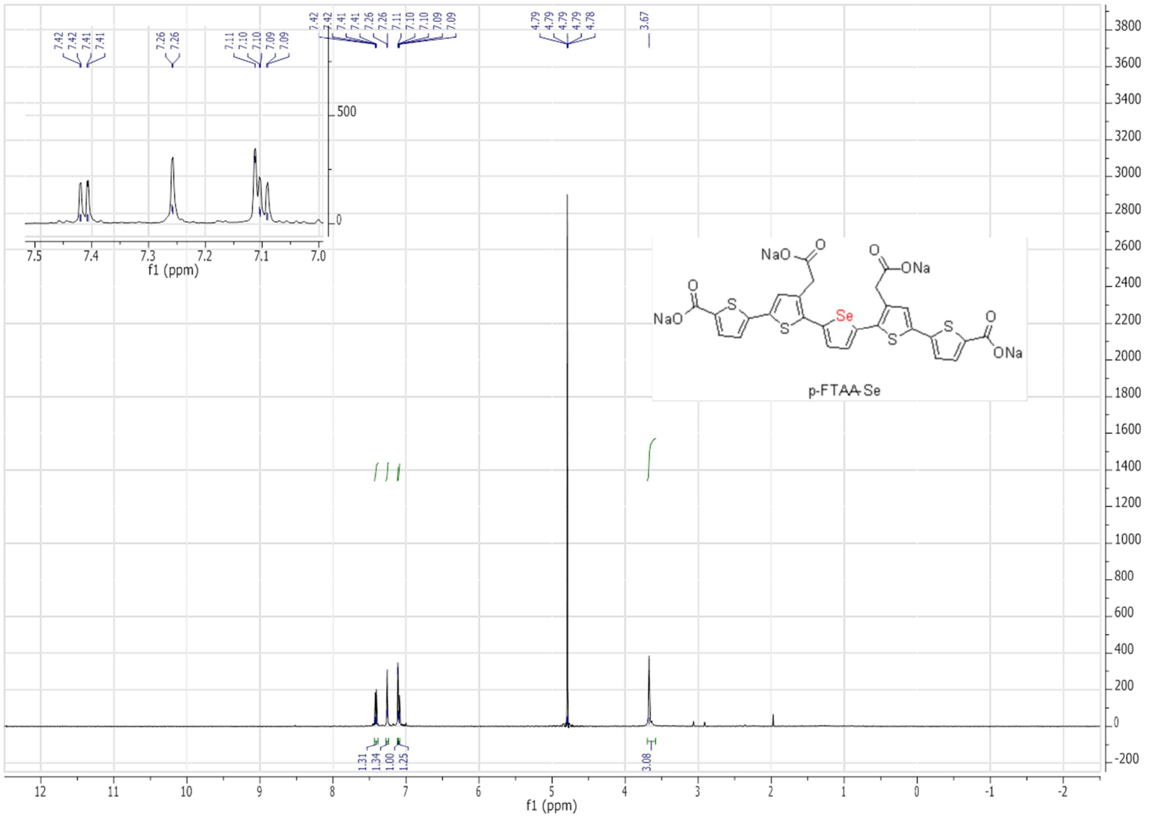

p-FTAA-Se
f1 (ppm)

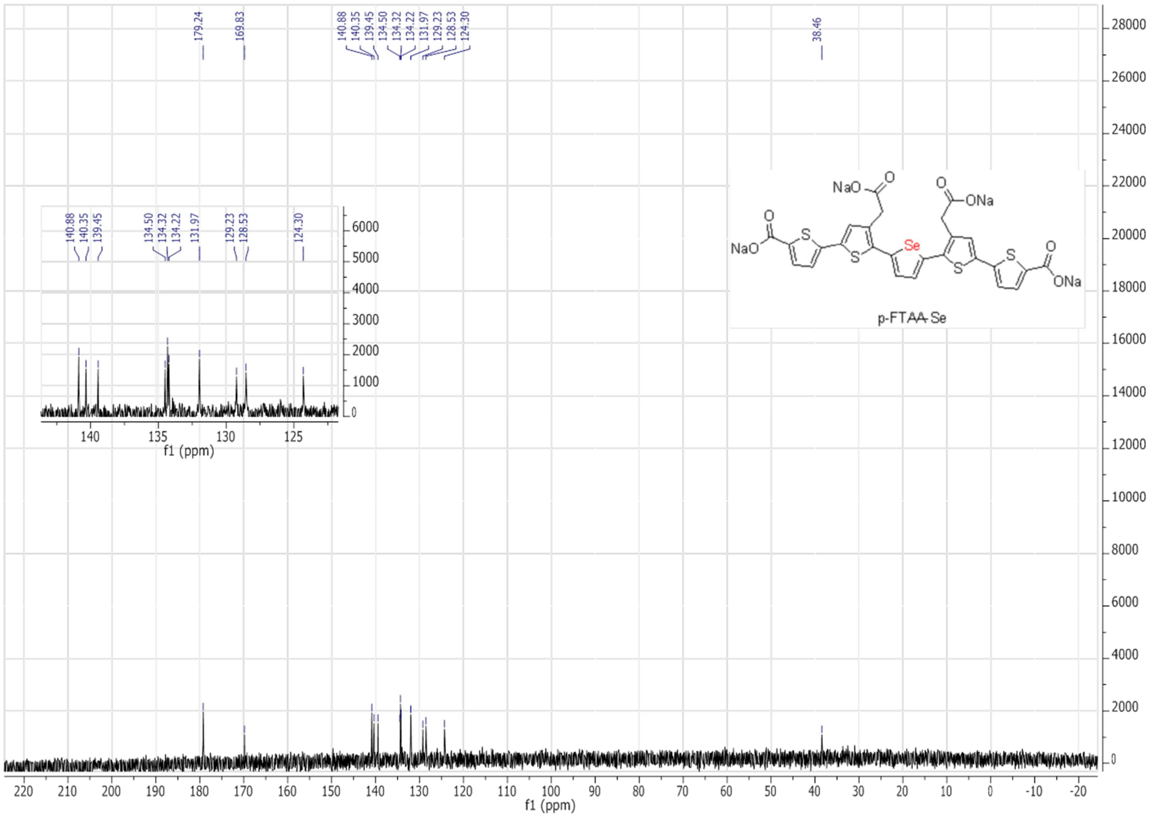

p-FTAA-Se
f1 (ppm)

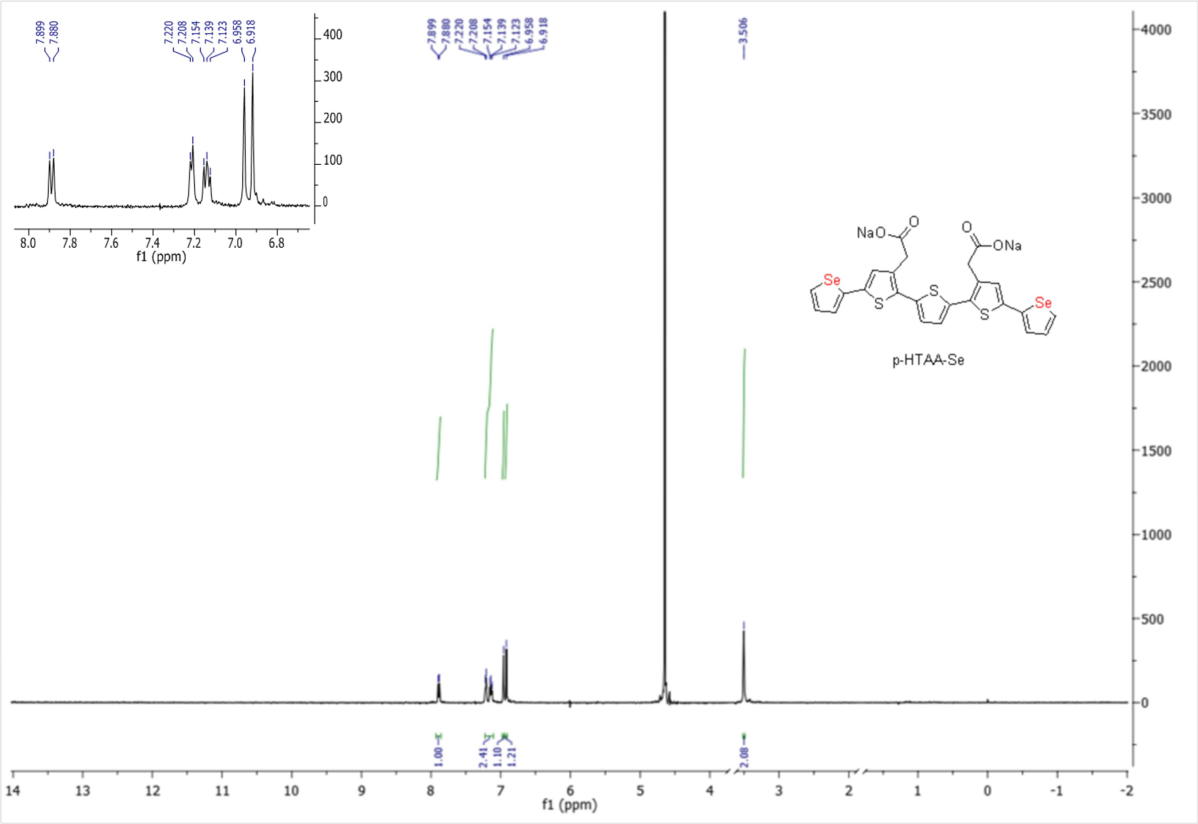
p-HTAA-Se
f1 (ppm)

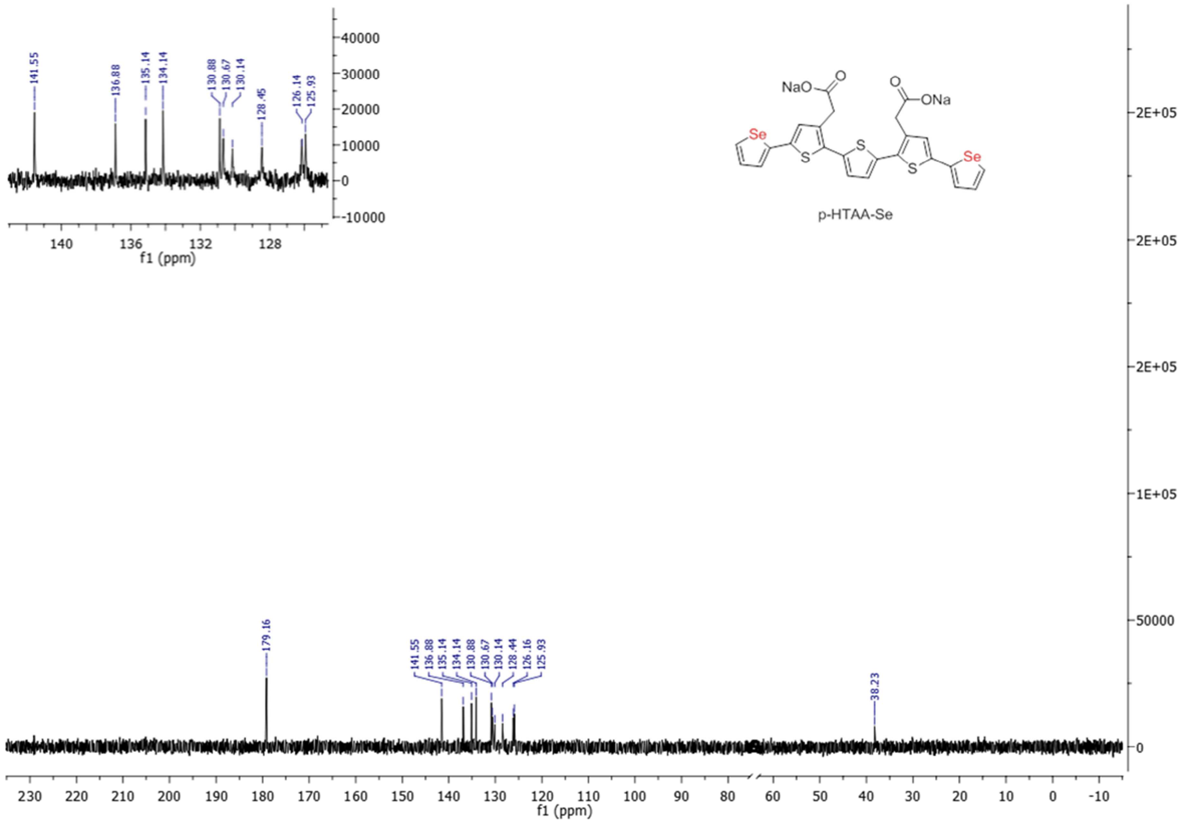
p-HTAA-Se
f1 (ppm)

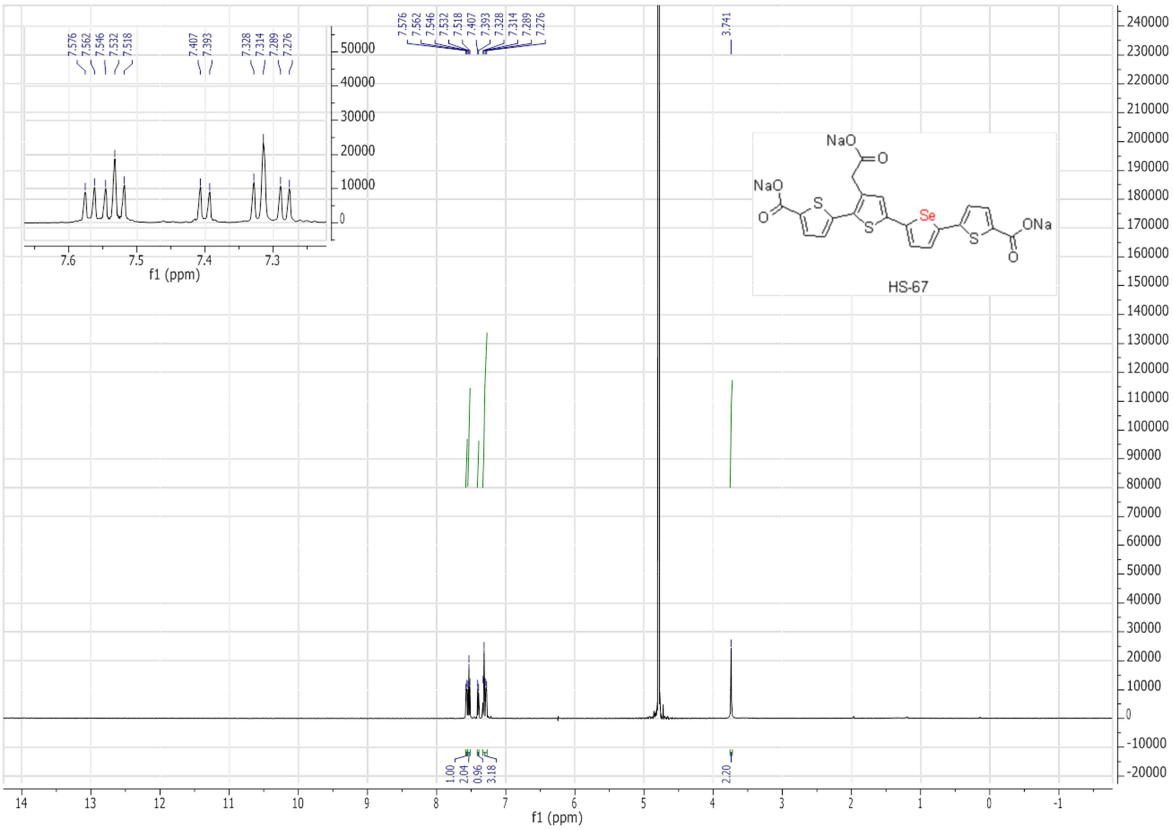
7.576 7.562 7.546 7.532 7.518 7.407 7.393 7.328 7.314 7.289 7.276
3.741
NaO
O
NaO
O
S
S
Se
S
ONa
O
HS-67
1.00 2.04 0.96 3.18
2.20
f1 (ppm)

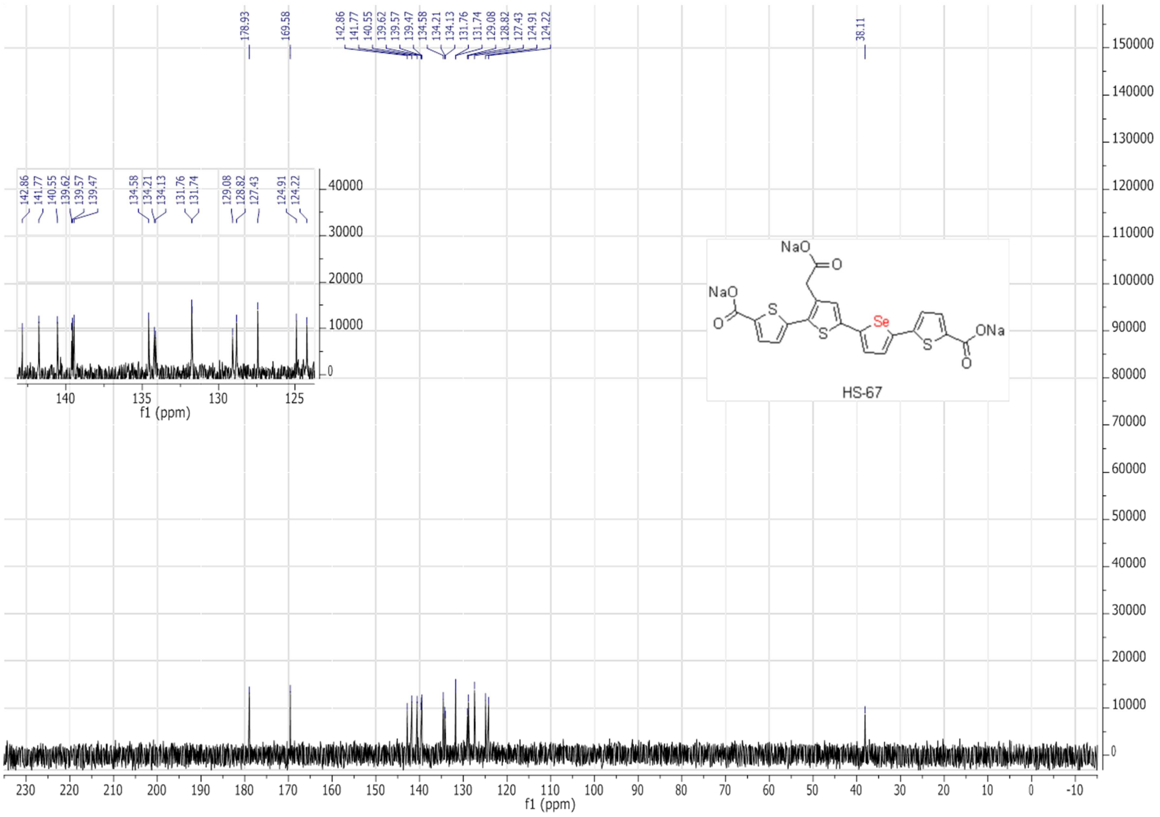
178.93
169.58
142.86 141.77 140.55 139.62 139.57 139.47 134.58 134.21 134.13 131.76 131.74 129.08 128.82 127.43 124.91 124.22
38.11
NaO
O
NaO
O
S
S
Se
S
ONa
O
HS-67
f1 (ppm)

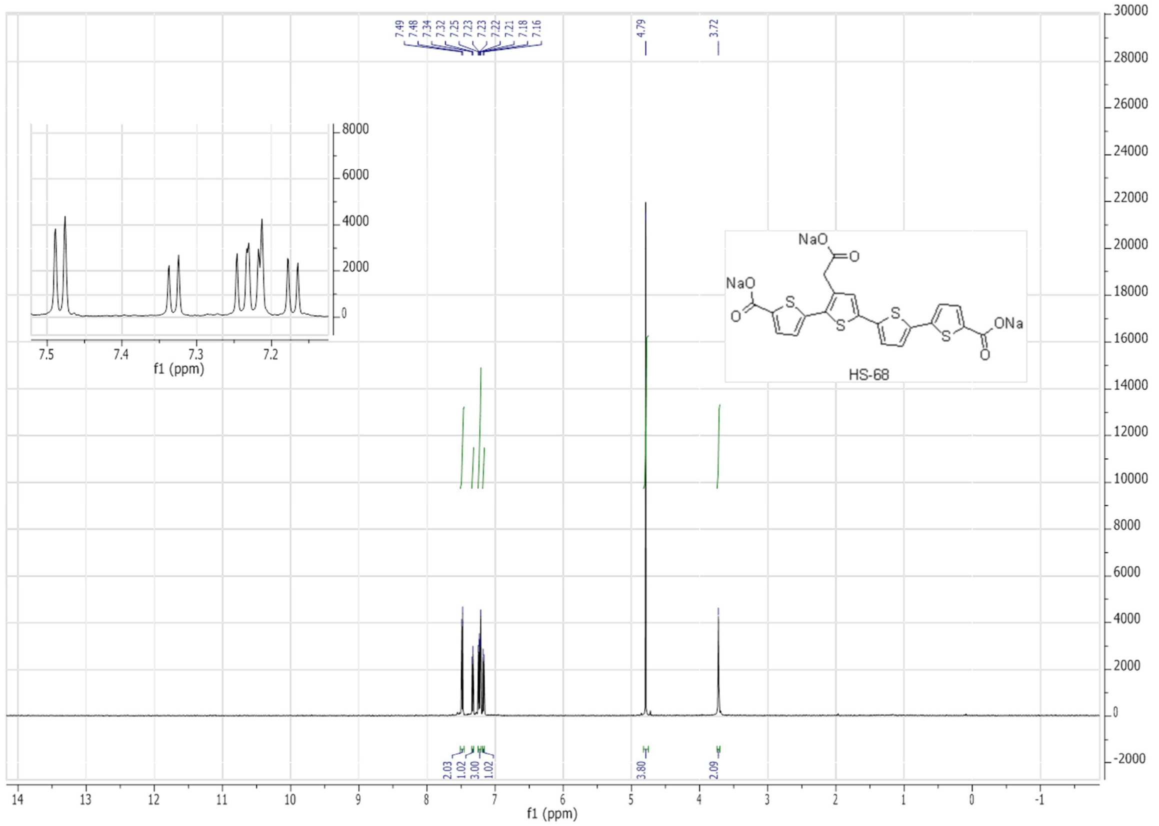
7.49 7.48 7.34 7.32 7.25 7.23 7.23 7.22 7.21 7.18 7.16
4.79
3.72
NaO
O
S
ONa
HS-68
2.03 1.02 3.00 1.02
3.80
2.09
f1 (ppm)

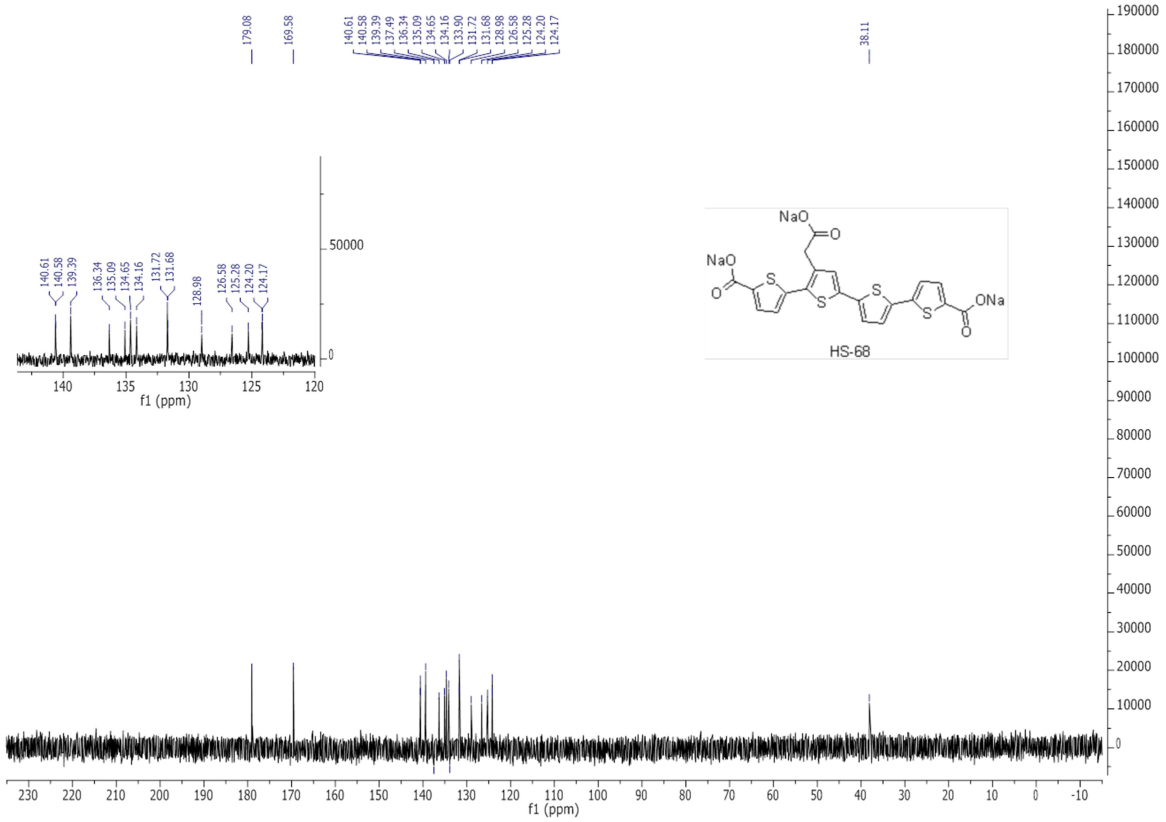
179.08
169.58
140.61 140.58 139.39 137.49 136.34 135.09 134.65 134.16 133.90 131.72 131.68 128.98 126.58 125.28 124.20 124.17
38.11
140.61 140.58 139.39 136.34 135.09 134.65 134.16 131.72 131.68 128.98 126.58 125.28 124.20 124.17
NaO
O
S
ONa
HS-68
f1 (ppm)

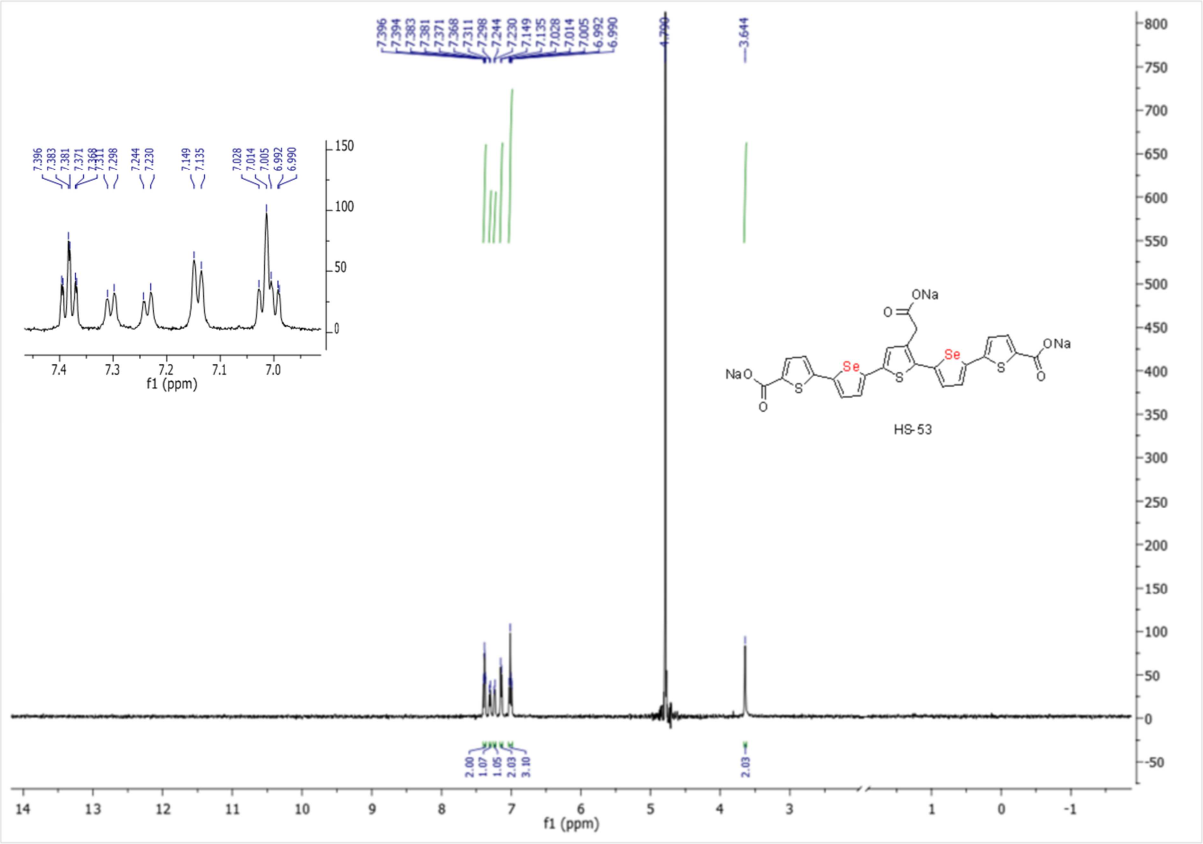
ONa
O
NaO
O
ONa
Se
Se
S
S
S
HS-53
f1 (ppm)

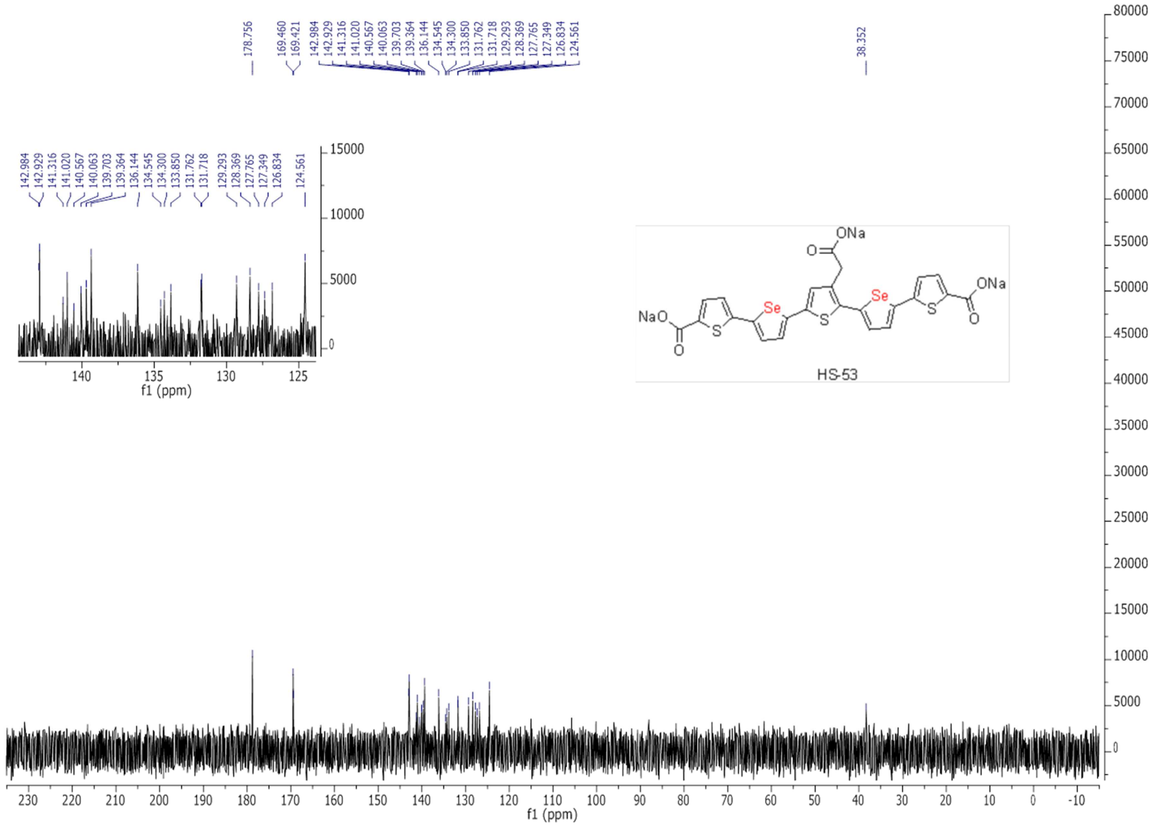
ONa
O
NaO
O
ONa
Se
Se
S
S
S
HS-53
f1 (ppm)

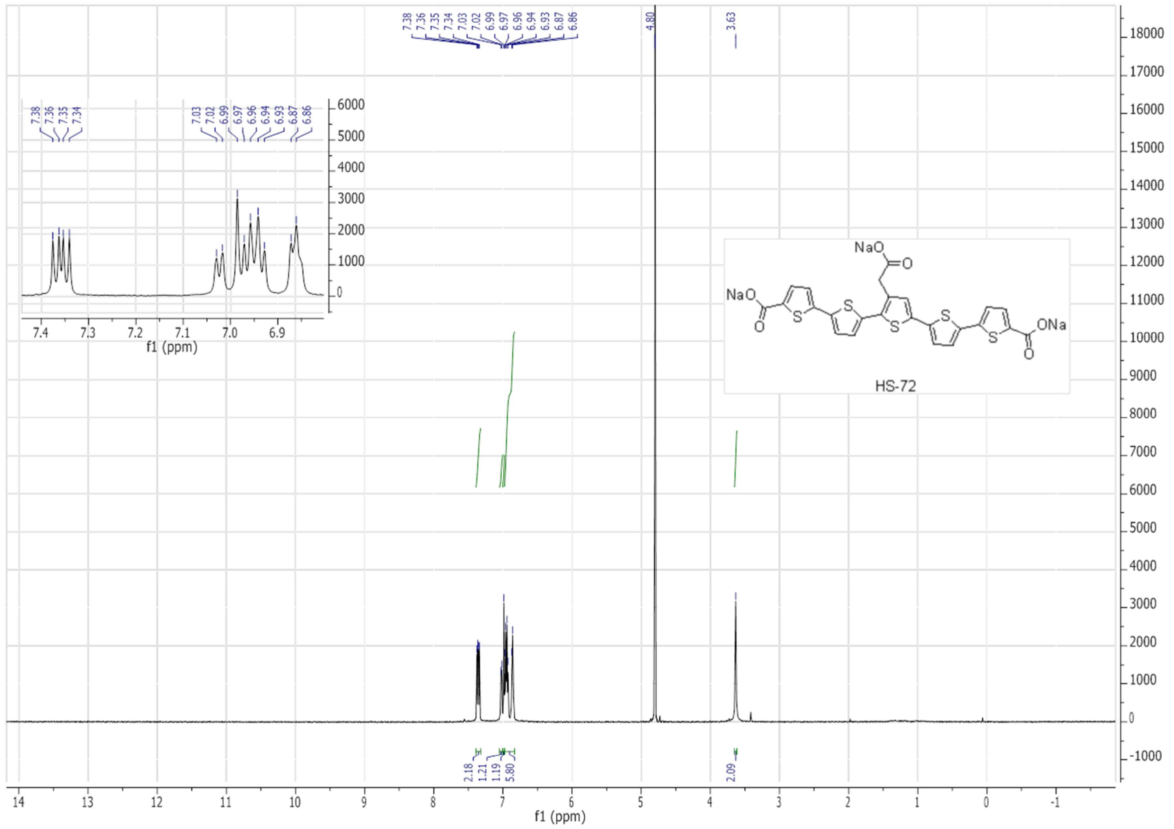
7.38
7.36
7.35
7.34
7.03
7.02
6.99
6.97
6.96
6.94
6.93
6.87
6.86
4.80
3.63
2.18
1.21
1.19
5.80
2.09
f1 (ppm)
NaO
O
NaO
ONa
HS-72

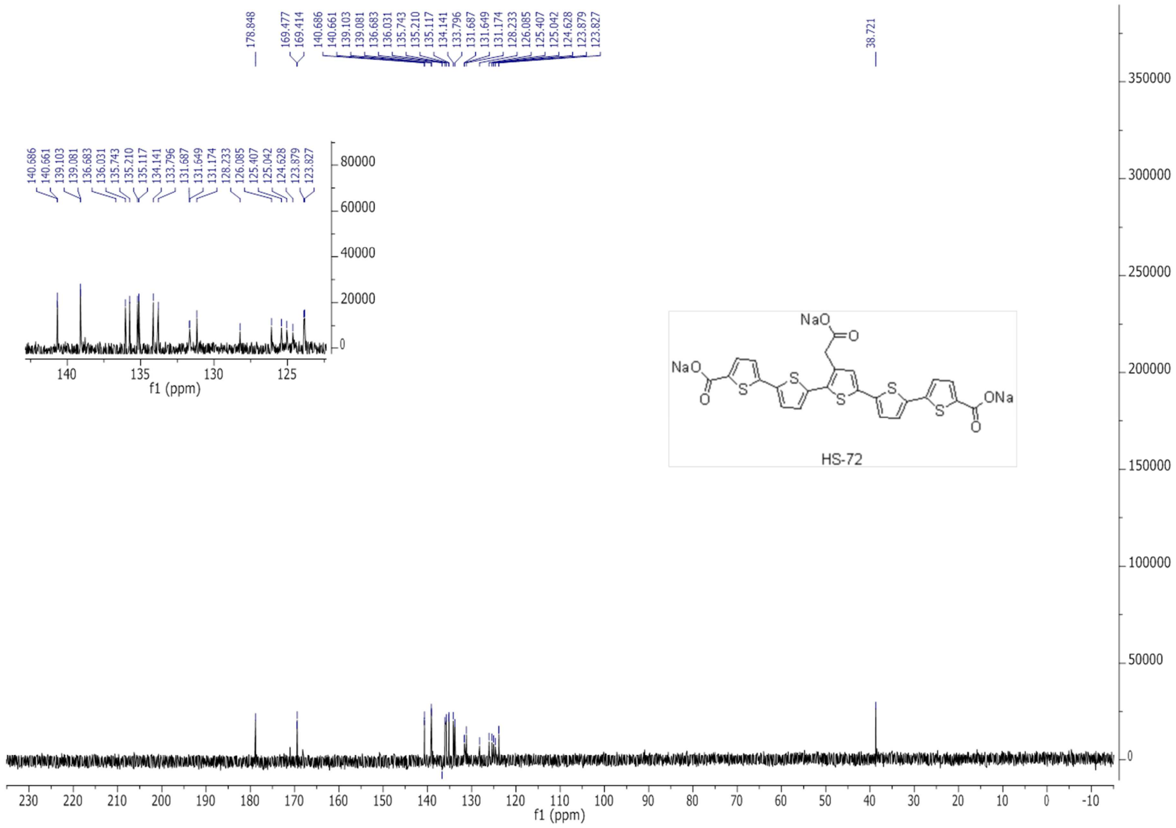
178.848
169.477
169.414
140.686
140.661
139.103
139.081
136.683
136.031
135.743
135.210
135.117
134.141
133.796
131.687
131.649
131.174
128.233
126.085
125.407
125.042
124.628
123.879
123.827
38.721
f1 (ppm)
NaO
O
NaO
ONa
HS-72

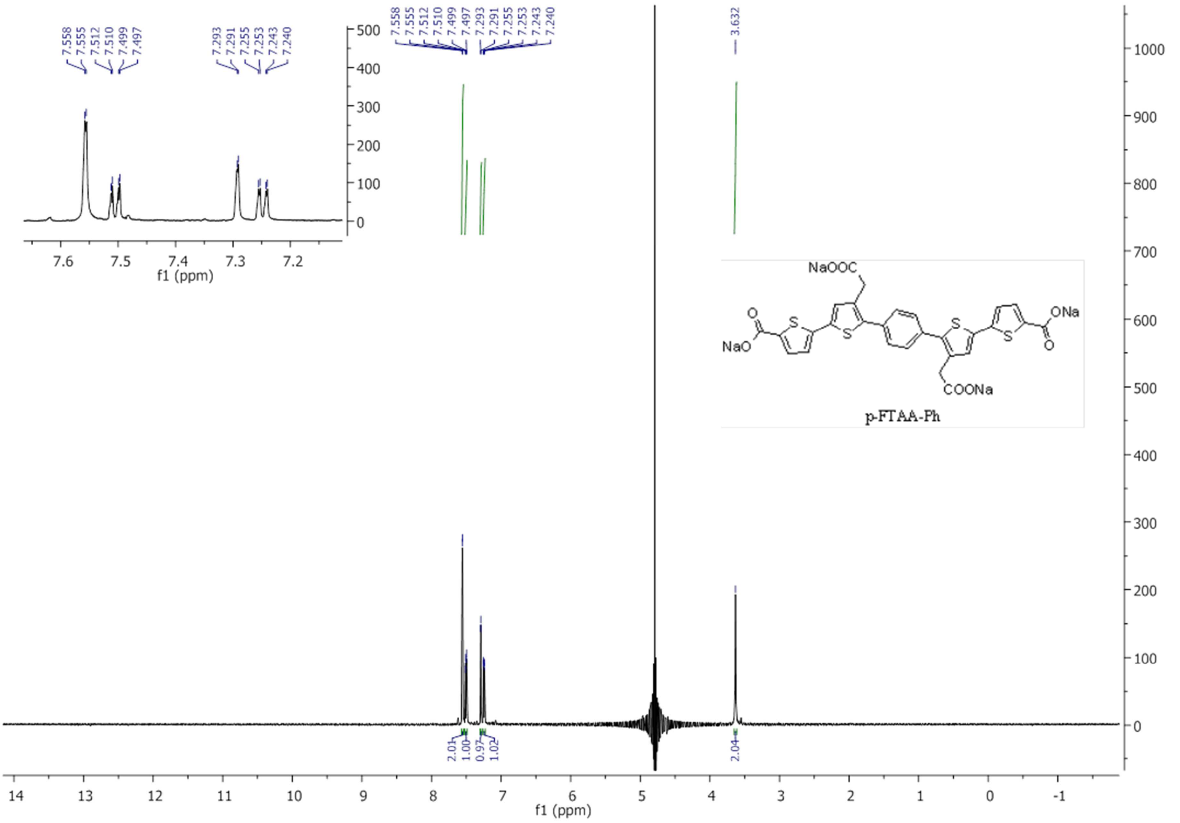
7.558
7.555
7.512
7.510
7.499
7.497
7.293
7.291
7.255
7.253
7.243
7.240
3.632
2.01
1.00
0.97
1.02
2.04
f1 (ppm)
NaOOC
ONa
NaO
COONa
p-FTAA-Ph

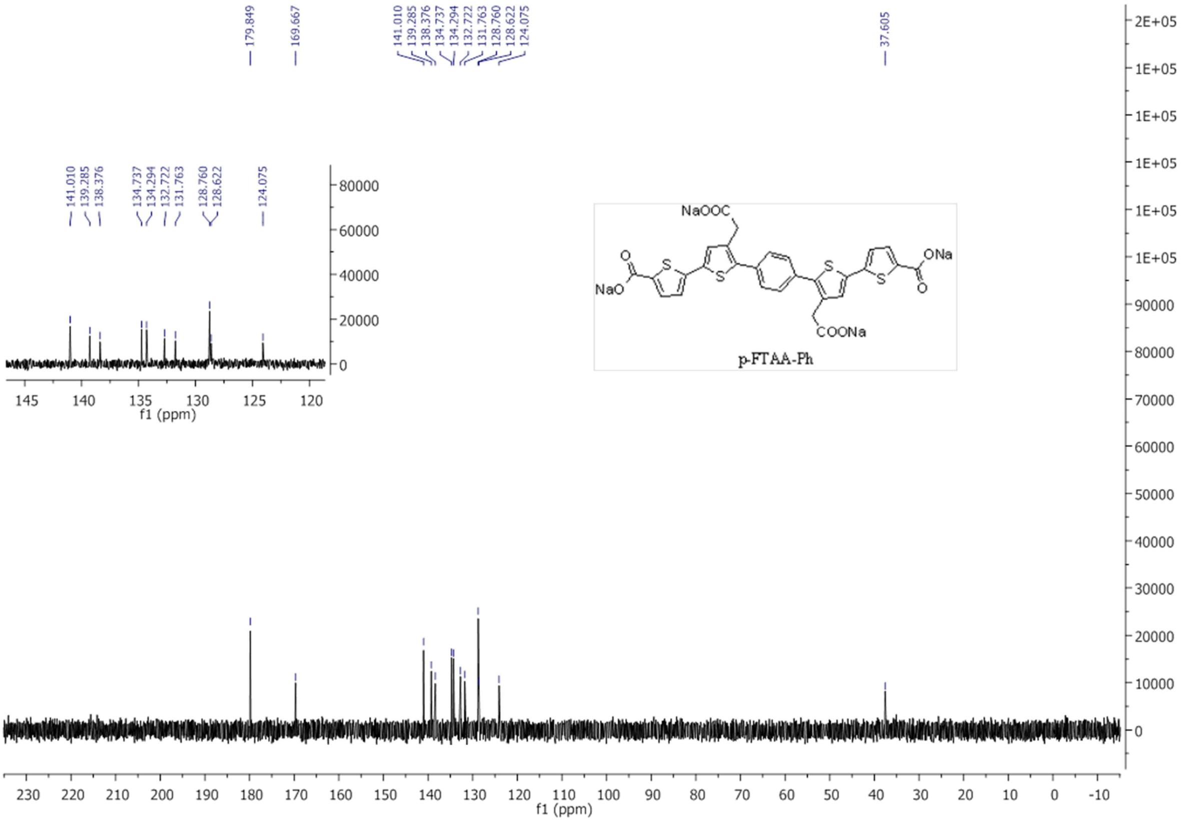
179.849
169.667
141.010
139.285
138.376
134.737
134.294
132.722
131.763
128.760
128.622
124.075
37.605
f1 (ppm)
NaOOC
ONa
NaO
COONa
p-FTAA-Ph

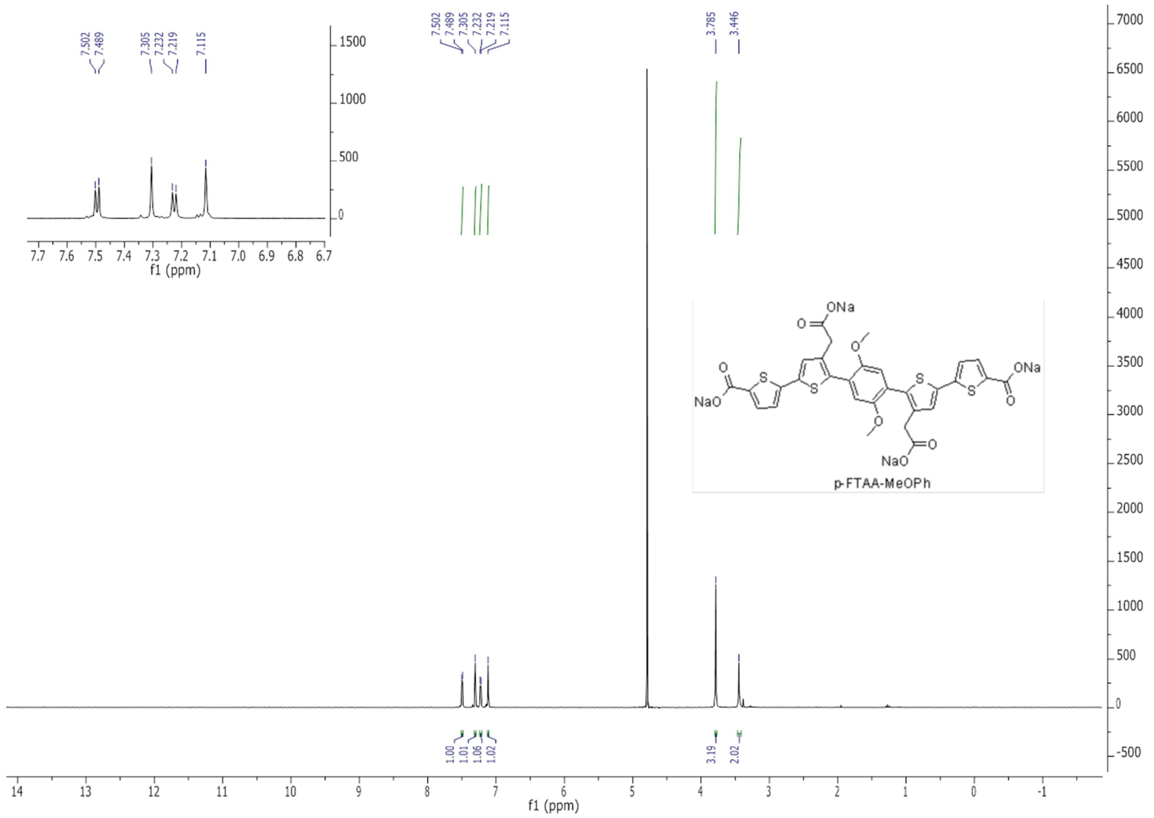
7.502
7.489
7.305
7.232
7.219
7.115
3.785
3.446
1.00
1.01
1.06
1.02
3.19
2.02
ONa
O
NaO
p-FTAA-MeOPh
f1 (ppm)

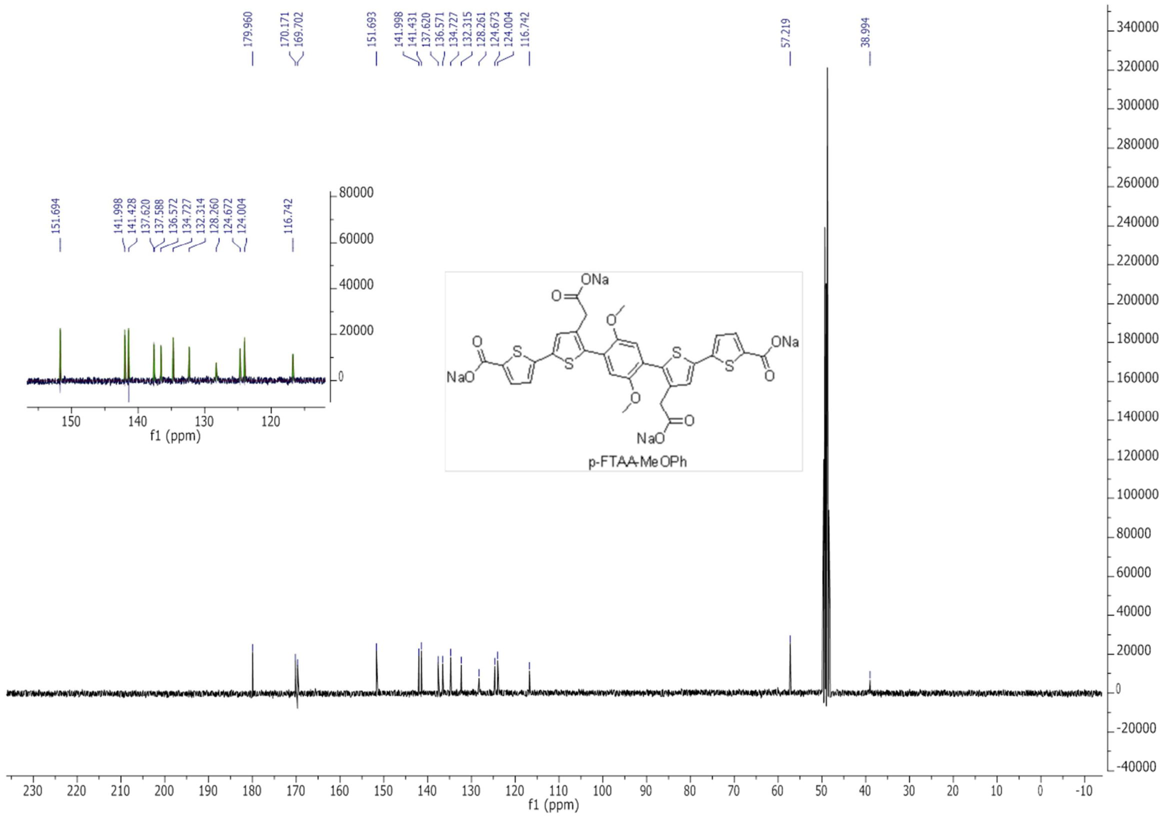
179.960
170.171
169.702
151.693
141.998
141.431
137.620
136.571
134.727
132.315
128.261
124.673
124.004
116.742
57.219
38.994
ONa
O
NaO
p-FTAA-MeOPh
f1 (ppm)
